# Exploring the structural variability in developing wheat grains using autofluorescence multispectral imaging at the macroscopic scale

**DOI:** 10.3389/fpls.2025.1580426

**Published:** 2025-06-19

**Authors:** Anne-Laure Chateigner-Boutin, Fabienne Guillon, Marie-Françoise Devaux

**Affiliations:** UR1268 BIA, INRAE, Nantes, France

**Keywords:** wheat grain, autofluorescence, multispectral imaging, large PCA, cell walls

## Abstract

**Introduction:**

The wheat grain undergoes major changes in dimensions, shape and composition during its development, and the outer tissues of the grain contribute to these modifications. Exploring the spatial and temporal variability in the developing wheat grain contributes to our knowledge of cereal grain development which is necessary to improve traits of interest such as grain weight and shape.

**Methods:**

A series of 40 autofluorescence multispectral images was acquired for whole grain sections at four developmental stages. The images were analyzed using principal component analysis of large image series (large PCA). This chemometric method enables the quantification of the variability, its localization, highlighting the associated spectral characteristics and their attribution to chemical compounds.

**Results:**

Regional and temporal heterogeneity was revealed. Among the new information, the lignification of cell walls in pericarp tissues first appeared patchy and later homogeneous. Polar differences have been found in aleurone cell walls for phenolic compounds.

**Discussion:**

This work demonstrates that multispectral autofluorescence analysis of plant organ sections combined with large PCA is a powerful tool for studying the relationship between tissue chemical composition, its variability and the internal structure of organs at the macroscopic scale.

## 1 Introduction

Wheat, the second most-produced cereal crop in the world, is mainly targeted for food and animal feed. Wheat production is facing several challenges, including the stagnation of grain yield while the global population, and hence demand, increases. One possible strategy to overcome yield stagnation is to increase the final weight of individual grains. In addition, the shape of the wheat kernel, with its distinctive deep crease, is a feature that influences milling performance. As grain dimensions, weight, and shape are related to grain growth, a thorough knowledge of the wheat grain development is necessary to improve both grain weight and shape.

Botanically, the wheat grain is a caryopsis, a dry fruit containing a unique seed. It is composed of an embryo and a storage tissue named endosperm, surrounded by several outer tissues called the nucellar epidermis, testa, and pericarp. The pericarp tissues comprise the epicarp, the outermost cell layer of the pericarp, the mesocarp, and the endocarp, also called the inner pericarp. Cuticles are present on the outer cell wall of the epicarp, nucellar epidermis, and testa. The crease area of the grain has a complex shape and structure. It comprises the same outer tissues as in the other regions of the grain, with a discontinuity in the endocarp and testa layers in which lies the pigment strand, beneath the nucellar projection and the apoplastic cavity, and above the central vascular tissue (Xiong et al., 2013; Chateigner-Boutin et al., 2021).

During the first phase of development, the grain grows mainly in length, and then it grows in volume while storage compounds accumulate in the endosperm. At the end of the filling stage, the grain’s maximal dimensions are reached, and desiccation occurs. At the tissue level, the grain development comprises steps of cell division, differentiation, and maturation, which do not occur simultaneously in all grain tissues. At the beginning of grain development, the pericarp occupies most of the grain. After a few days, the mesocarp undergoes developmentally programmed cell death, leaving space for the developing endosperm and embryo. The embryo and endosperm cells first divide and then differentiate, with the latter giving rise to the starchy endosperm and its surrounding aleurone layer. The nucellar epidermis and testa differentiate between the pericarp and endosperm, followed by cell lysis, and at the mature stage, only their cell walls remain (Evers and Millar, 2002; Chateigner-Boutin et al., 2018).

The grain outer tissues contribute to the determinism of grain length and shape since at the stages when these traits are set, grains are mostly composed of pericarp. Moreover, several studies have suggested that the grain outer tissues contribute to limiting grain size and weight by constraining grain growth. Differences in elasticity/stiffness of tissues due to differences in cell wall composition, cross-linking state, and properties may contribute to differential growth and growth cessation (Peaucelle et al., 2011; Dauphin et al., 2022). In detail, cell walls are made up of a complex network of intertwined polymers, the main components of which are cellulose, hemicelluloses, lignin, and pectin. The composition of the cell wall depends on the tissue, the stage of development, and the environmental conditions.

A comprehensive study of cell wall heterogeneity in the outer tissues of the developing grain is needed, and this requires exploring both spatial and temporal variability. To this end, chemical mapping using spectral imaging techniques such as mid-infrared or Raman microspectroscopy can be used (Barron et al., 2005; Guillon et al., 2022). Fluorescence microscopy was also applied to map the distribution of phenolic compounds such as lignin and hydroxycinnamic acids in the cell walls of wheat grains. In this case, multiple bandpass excitation/emission filters or a few excitation wavelengths together with spectral detectors have been used to obtain multispectral or hyperspectral autofluorescence images (Courcoux et al., 2002; Ghaffari et al., 2019). All these techniques are limited in terms of field of view and are low throughput, not allowing the analysis of large numbers of samples. To cope with biological variability, experiments need to be repeated and therefore include many samples. In addition, the macroscopic study of entire organ sections would be of great help in the identification of key tissues and regions of the grain and key stages for the chemical modification of cell walls in these tissues.

Fluorescence macroscopy can combine a large field of view with good spatial resolution (below 3 µm per pixel), thus enabling the observation of an entire organ section of several mm^2^ with resolved histological structures (Devaux et al., 2023). With no prior sample preparation and fast acquisition time, it can be used to compare several samples. With the possibility of acquiring mosaic images, entire organ sections can be easily acquired. The technique was applied to mature wheat grain sections, allowing the distinction of pericarp, aleurone, and starchy endosperm tissues (Corcel et al., 2016).

In the present work, we propose to use full-field autofluorescence macroscopy to study the ontogeny of the outer tissues of wheat grains during their development at the scale of the entire grain section, taking grain variability into account. Four key developmental stages were compared on the basis of a series of 40 sections obtained from several wheat grains. The fluorescence macroscope was equipped with filters for acquiring color images after excitation in the UV and visible range, resulting in a multispectral image with 12 fluorescence emission channels. Each wheat grain image was a large multispectral image containing more than 3500 × 2500 pixels × 12 fluorescence values. The analysis of the large multispectral image series required the implementation of an analysis protocol, including all images and the calculation of indicators for the quantification of differences between images and developmental stages.

We propose here to apply principal component analysis (PCA) as the core technique capable of highlighting the most contrasting pixels. In the present case, the amount of data to be processed together was considerable, and conventional computers and software cannot handle the volume of data obtained by simply concatenating pixels. To manage and analyze a series of large images together in a consistent way, Devaux et al. (2023) adapted the method, called large PCA, which consists in iteratively computing the variance–covariance matrix and extracting common loadings for the entire image series.

The expected results were to reveal the fluorescence characteristics of the different tissues of the wheat grain according to the stage of development, to assess their spatial and developmental variability, and to progress in the knowledge of the associated fluorescent compounds.

## 2 Materials and methods

### 2.1 Wheat grains

Bread wheat (hexaploid wheat, *Triticum aestivum* L.) plants (cultivar Recital) were cultivated at INRAE Le Rheu (France) in a glasshouse with temperature monitoring as described previously (Verhertbruggen et al., 2021). Grain spikes were tagged at flowering, and development was estimated by adding the average temperature in Celsius degrees per day after flowering (°DAF). Wheat grains were collected at four key developmental stages, with 250°DAF corresponding to the end of grain elongation (maximum length reached, beginning of grain filling), 450°DAF corresponding to grain filling, 650°DAF corresponding to the end of filling (maximum volume reached), and 850°DAF corresponding to grain desiccation. Four to five– plants were considered per stage, and one spike was taken per plant. Given that grains at the same stage of development are located at equivalent positions within a spike, the two grains located at the basal position of the spikelets themselves, located in the middle of the spike, were harvested (see **Supplementary Figure 1**). Grains were collected at different dates corresponding to different stages of development. They were frozen in liquid nitrogen and stored at −80°C.

### 2.2 Wheat grain sections

Grains were cryo-sectioned in the middle part of the grain using a cryotome (HM 500 OM, Microm, Dreieich, Germany) to obtain transverse sections with a thickness of 20 µm. The direct cutting of the grains produced damaged sections. Therefore, to allow sectioning with reduced tissue damage, grains were surrounded by a freezing medium [tissue freezing medium (TFM), VWR, Radnor, PA, USA].

Sections were placed on quartz slides of 17-µm thickness. For each stage of development, sections were taken from five grains, with one to three sections retained per grain, resulting in a total of 10 sections analyzed per stage.

### 2.3 Image acquisition

The multispectral autofluorescence images were acquired using the fluorescence macroscope Multizoom AZ100M (Nikon, Tokyo, Japan) equipped with a QImaging EXi Aqua monochrome camera plus an RGB-HM-S-IR filter wheel for color image acquisition. The system provides 1392 × 1040 pixels RGB images with gray-level intensities coded using 2^14^ = 16,384 values. The total magnification was set to ×8 by combining the lens AZ-Plan Fluor 2X (NA: 0.2/WD: 45 mm) and a ×4 optical zoom. With these settings, the pixel size was 1.44 µm, and the field of view was 2.0 × 1.5 mm^2^. The macroscope was equipped with a Prior Proscan II (Nikon, Japan) motorized stage, allowing large image acquisition. The INTENSILIGHT (C-HGFI/C-HGFIE Precentered Fiber Illuminator, Nikon, Japan) device, including a mercury lamp, ensures lighting for fluorescence imaging. Four fluorescence excitation/emission filter cubes corresponding to two UV excitations (U1 and U2) and two visible [Blue (BL) and Green (GR)] excitations were placed inside the motorized filter wheel (**Supplementary Table 1**).

The acquisition software NIS-Elements (AR 5.02.02) allows automated multispectral acquisition of large images. In general, four images were necessary to observe and reconstruct the entire grain section. The multispectral sequence was designed to successively acquire the four RGB images corresponding to the four fluorescence filters for a given field of view before moving to the next field of view. The order of acquisition was GR, BL, U2, and U1, with exposure times set after observation of a few samples (**Supplementary Table 1**). The ND-acquisition procedure in the NIS-Element software enables large images to be built automatically after all individual image acquisitions. As, after all acquisitions, the fluorescence intensity was found to be much lower for the two visible filters compared to the UV ones, a multiplicative factor was applied to the RGB images of filters Blue and Green.

One large image was acquired for each grain section, resulting in a set of 40 multispectral large images.

### 2.4 Multispectral images and pseudo-spectra

**Figure 1:**
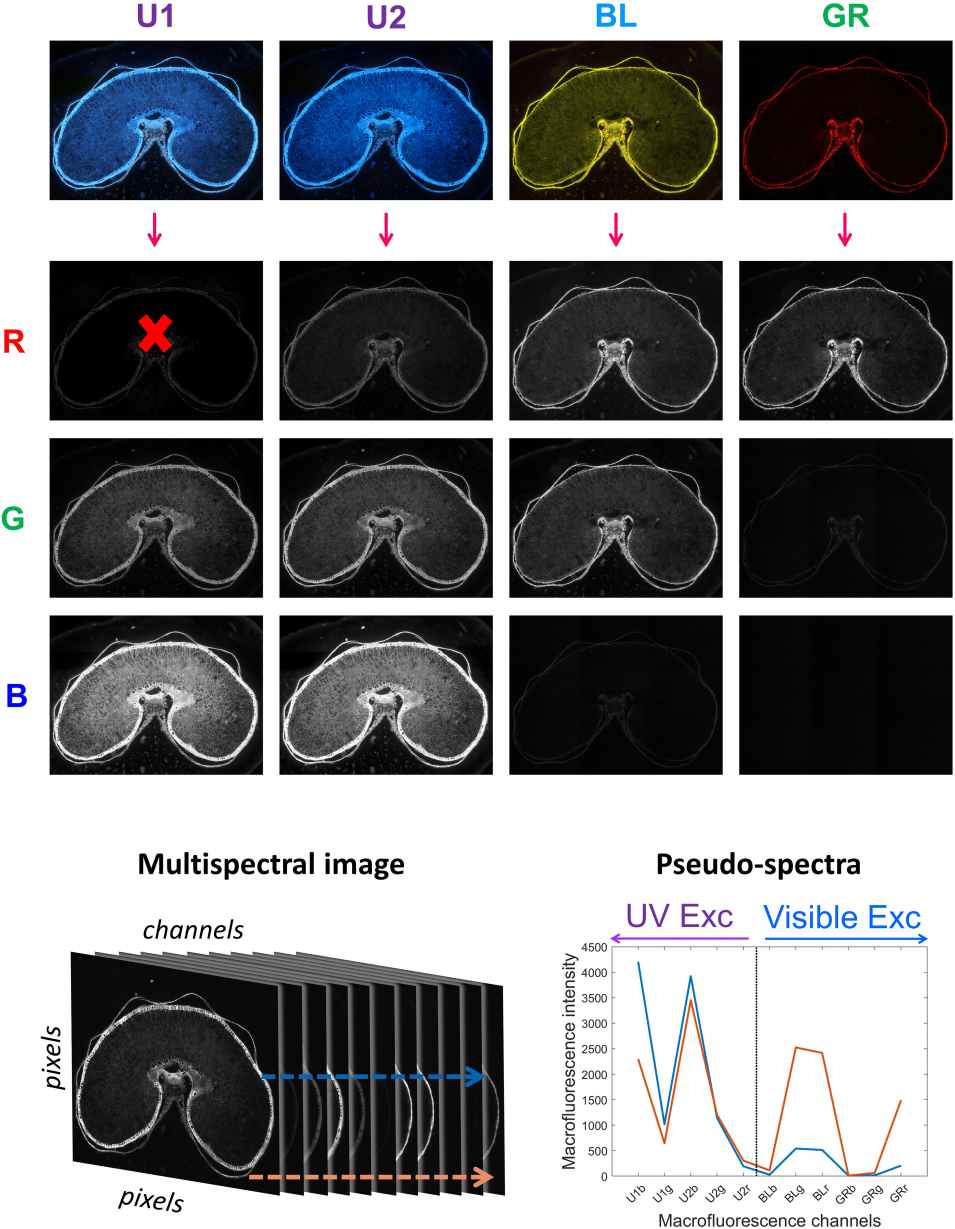
Building the multispectral image. One color RGB image is acquired for each filter: U1, U2, BL, and GR excitations. The red, green, and blue channels of the RGB images are considered. The red channel of U1 excitation is removed due to unwanted reflection. The multispectral image is built from the 11 selected fluorescence channels. Two examples of pseudo-spectra for pixels selected in the pericarp (orange) and in the aleurone layer (blue) are shown.

The multispectral images contained 12 channels by merging the four RGB images recorded using the filter cubes (**Figure 1**) (Corcel et al., 2016). The channels were placed in order from high to low wavelengths: blue, green, and red channels of each RGB image acquired with filters U1, U2, Blue, and Green. The channel names were U1b, U1g, U1r, U2b, U2g, U2r, BLb, BLg, BLr, GRb, GRg, and GRr. Channel U1r was removed from the sequence because it contained unwanted reflection from the excitation Rayleigh band. The final multispectral image, therefore, contained 11 channels.

For each pixel, 11 fluorescence intensity values were measured. The set of fluorescence intensities measured for individual pixels was called “pseudo-spectra” (**Figure 1**) (Corcel et al., 2016). The left and right parts of the pseudo-spectra correspond to the emission channels after UV (UV1 and UV2) and visible (Blue and Green) excitations, respectively. Because no photon can be emitted at wavelengths lower than the excitation wavelength, for the two visible excitations, Blue and Green, channels BLb, GRb, and GRg showed no signals. They were, nevertheless, maintained in the pseudo-spectra and were considered as a baseline.

### 2.5 Image processing

The analysis workflow (**Supplementary Figure 2**) included steps to process the multispectral images individually and steps to compare the series of 40 images and quantify the differences between the developmental stages.

Individual multispectral images:

- background subtraction and calculation of the image “sum of fluorescence intensity”,
- computing two regions of interest: grain sections and outer grain tissues, and
- computing RGB composite fluorescence images to provide a visual overview of the differences between the series of multispectral images.

Analysis of the series of 40 images

- PCA of the series using the adaptation of PCA to large image series,
- computing score distributions for the pixels of the outer grain tissues, and
- comparing score distributions by a usual PCA to highlight the significant differences between developmental stages.

All the image processing and data processing were performed in the Matlab 2022a environment (The MathWorks, Natick, MA, USA) using the Image Processing Toolbox, the Statistics and Machine Learning Toolbox, dedicated homemade functions, and scripts developed for collections of macrofluorescence multispectral images.

#### 2.5.1 Image preprocessing

A preliminary analysis revealed a channel-dependent background intensity. Three regions without any signal were manually selected in four images of the series. The background intensity of each channel was calculated as the average intensity of the 12 regions for that channel. The values were subtracted from the corresponding images for all images.

#### 2.5.2 Multispectral image representation

A visual comparison of multispectral images is not straightforward. For this purpose, RGB composite macrofluorescence images were computed as described in Devaux et al. (2023). The RGB composite image corresponds to the average red, green, and blue channels of the color image acquired for the four filters, avoiding channels BLb, GRb, and GRg, which showed no signals.

The RGB composite images were computed after background subtraction using a common min–max of 0 and 11,000 fluorescence counts and a gamma coefficient of 0.5 to enhance the low fluorescence intensities.

One gray-level image was also computed for segmentation purposes as the “sum of fluorescence intensity” of the 11 channels of the multispectral image. The image was converted into 256 gray levels.

#### 2.5.3 Image analysis

##### 2.5.3.1 Segmentation of the regions of interest of the whole grain sections

Pixels corresponding to the grain section were segmented from the image “sum of fluorescence intensity”. A common gray-level threshold value of 2 was used for all images. The resulting regions were postprocessed by morphological opening and closing using squared structuring elements (Soille, 2003) with sizes 51 and 150 pixels, respectively. For three images, the threshold was not appropriate and was set individually. In addition to being used for further analysis, the section regions of interest were also used to measure the surface area of the grain sections.

##### 2.5.3.2 Segmentation of the regions of interest of the grain outer tissues

Pixels corresponding to the outer tissues were segmented as follows. A first Otsu threshold (graythresh.m Matlab function) (Otsu, 1979) was computed from the pixels within the whole grain sections using the “*sum of fluorescence intensity*” image. A second Otsu threshold was computed using the sum of intensities of channels U1b, U1g, U2b, U2g, and BLg, avoiding red emission fluorescence. Both thresholds may have been adjusted after user checking. The two resulting regions were merged to obtain the raw outer tissue region of interest. The region was postprocessed via morphological operators, using disks as structuring elements: removing noise by opening of size 1, enlarging the region by dilation of size 8, and openings of size 12 to remove irregularities of contours. The size of morphological operators may have been adjusted after user checking. The largest segmented region was finally selected as the outer tissue region of interest. Regions of interest can be visualized in the **Supplementary Material** (supplementaryRoiOuterTissues).

##### 2.5.3.3 Principal component analysis of a series of large multispectral images

PCA is the basic method applied to multispectral image analysis (Geladi and Grahn, 1996). It consists in considering the images as sets of pixels, i.e., sets of pseudo-spectra, therefore ignoring the spatial nature of the multispectral images. PCA is applied to the set of pseudo-spectra, and loadings and scores are computed. Scores can be refolded to be visualized as score images. They reveal the regions of the images where the pixels contrasted by the components are located. Loadings provide spectral interpretation.

PCA has been adapted to analyze a series of large images called “large PCA” (Devaux et al., 2023). The main steps are recalled here.

In the large PCA software, all images are considered iteratively. Each multispectral image is unfolded in the form of a usual data table **X*_i_***. Local values are computed from this data table: the number of pixels (i.e. the number of pseudo-spectra in the multispectral image **X*_i_***); the sum of all pseudo-spectra as a local contribution to the global mean spectra; and the local contribution to the total variance–covariance matrix (**X*_i_*** ′**X*_i_***, where **X*_i_*** ′ denotes the transpose of **X*_i_***). These local values are used to compute the total number of pixels included in the analysis, the mean pseudo-spectra of all the pixels, and the global variance–covariance matrix. Eigenvalues and loadings **L** are obtained by the singular value decomposition of the global variance–covariance matrix. The loadings are common to all images in the series. The scores are calculated from the loadings **L** by iteratively re-considering each image after subtracting the global mean pseudo-spectra. The scores are finally refolded to form score images called large PCA scores (**Supplementary Figure 2**).

The representation of scores as images is obtained as described in Devaux et al. (2023). Intensities were converted to 8 bits by calculating common minimum and maximum values from the eigenvalues of the large PCA. This common grayscale allows the comparison of scores from one image to another.

In the present work, large PCA was applied on the pixels of the 40 multispectral images corresponding to the whole section regions of interest.

##### 2.5.3.4 Distributions of large PCA scores

The principal component scores are represented in the form of images, enabling a visual comparison of the different sections. A quantitative comparison of multispectral images was obtained by calculating, for each component, the observed distribution of pixels (Devaux et al., 2023). The principle is described here. First, score distributions were calculated for each principal component by considering all the pixels in the series of images (**Supplementary Figure 3**). Considering the huge number of pixels (more than 240 million), a large number of bins was *a priori* set to 10,000 with linear edges between each bin. In a second step, percentiles of the resulting distribution were computed (**Supplementary Figure 3**). These percentiles define new bins with variable size. Finally, the new bins were used to calculate the observed score distributions for each multispectral image.

This quantification was justified by the fact that large PCA reveals pixels with contrasting spectral properties. Calculating score distributions using bins common to the series of images is one way of revealing the images in which these pixels are found.

In the present work, the percentiles of the global distributions were set to 0 to 100 per step of 1%. The observed score distributions were computed by considering only the pixels belonging to the regions of interest corresponding to the grain outer tissues.

##### 2.5.3.5 Analysis of the large PCA score distributions

Average score distributions were computed according to the wheat developmental stages, and PCA was applied to the series of score distributions.

## 3 Results and discussion

### 3.1 Tissues identified in wheat grain cryo-sections at the macroscopic scale

**Figure 2:**
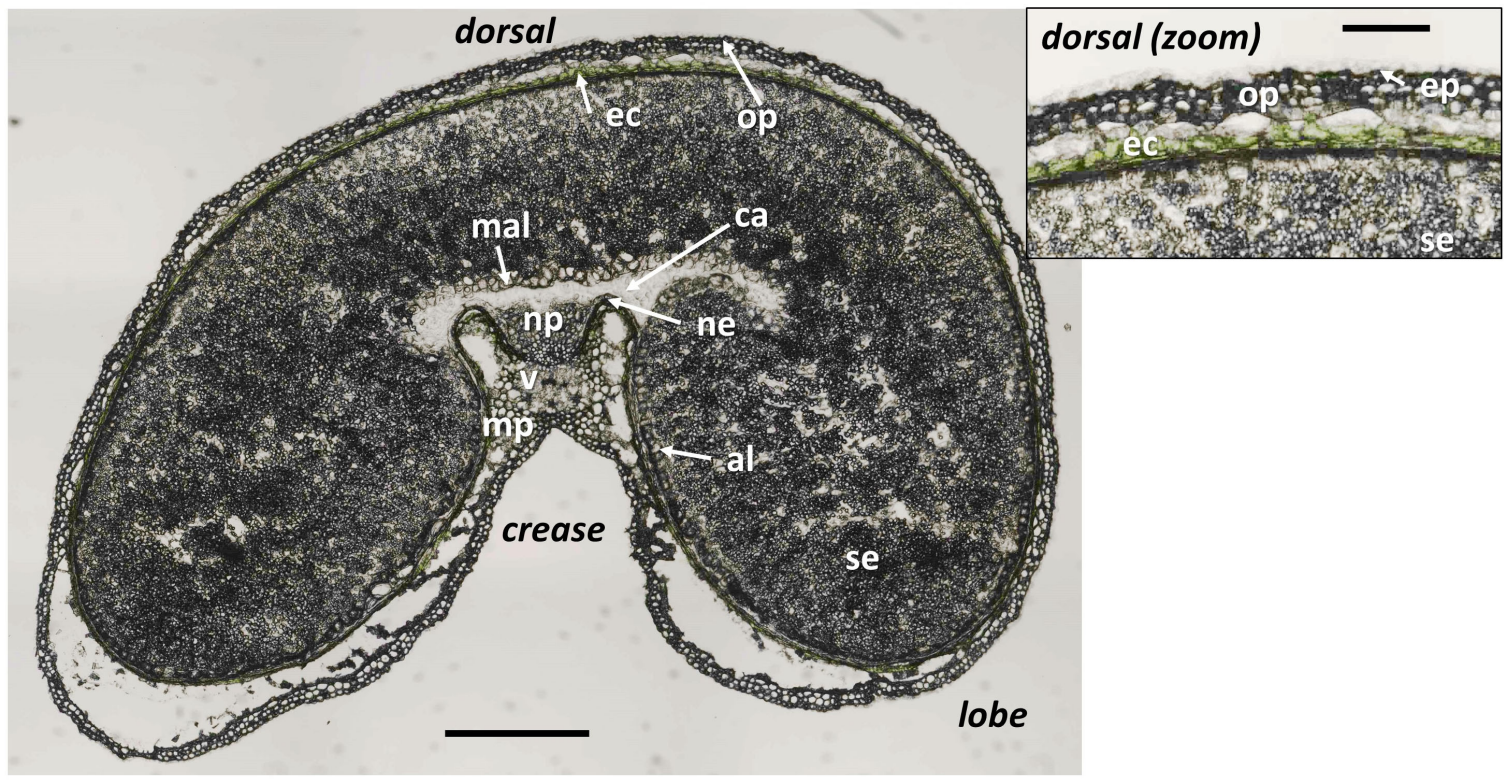
Tissues identified in the wheat cryo-sections at the macroscopic scale. Brightfield image of a wheat grain transverse cryo-section at stage 250°DAF. Ep, epicarp; mp, mesocarp; op, outer pericarp; ec, endocarp; ne, nucellar epidermis; al, aleurone; se, starchy endosperm; mal, modified aleurone; np, nucellar projection; v, vascular tissue; ca, apoplastic cavity. Scale bar represents 1.5 mm and 500 µm in the magnified image.

**Figure 2** shows an example of a macroscope brightfield image of a grain section at 250°DAF acquired with the same spatial resolution as the fluorescence images. Tissues were identified by comparison with images obtained with higher magnification on thin sections of fixed and resin-embedded grains (Chateigner-Boutin et al., 2018). The sections were obtained from the equatorial part of the grain, which was located above the embryo and scutellum, explaining why they were not visible in the sections.

In such an equatorial section, the central starchy endosperm occupies the inside of the grain and is surrounded by the outer layers. At 250°DAF, the aleurone layer is only visible in the crease region. Above it, there is a thick green layer, which corresponds to the endocarp containing chloroplasts, and in between, the dark line corresponds to the testa and nucellar epidermis, which cannot be distinguished. Several cell layers are detached from the rest of the grain. These tissues are composed of mesocarp cells and epicarp cells, and for the rest of the manuscript, will be referred to as the outer pericarp. In the crease area, near the vascular tissue, the mesocarp that contains chloroplasts is visible, as well as the nucellar projection and the apoplastic cavity.

### 3.2 RGB composite macrofluorescence images reveal spatial and developmental variability

**Figure 3:**
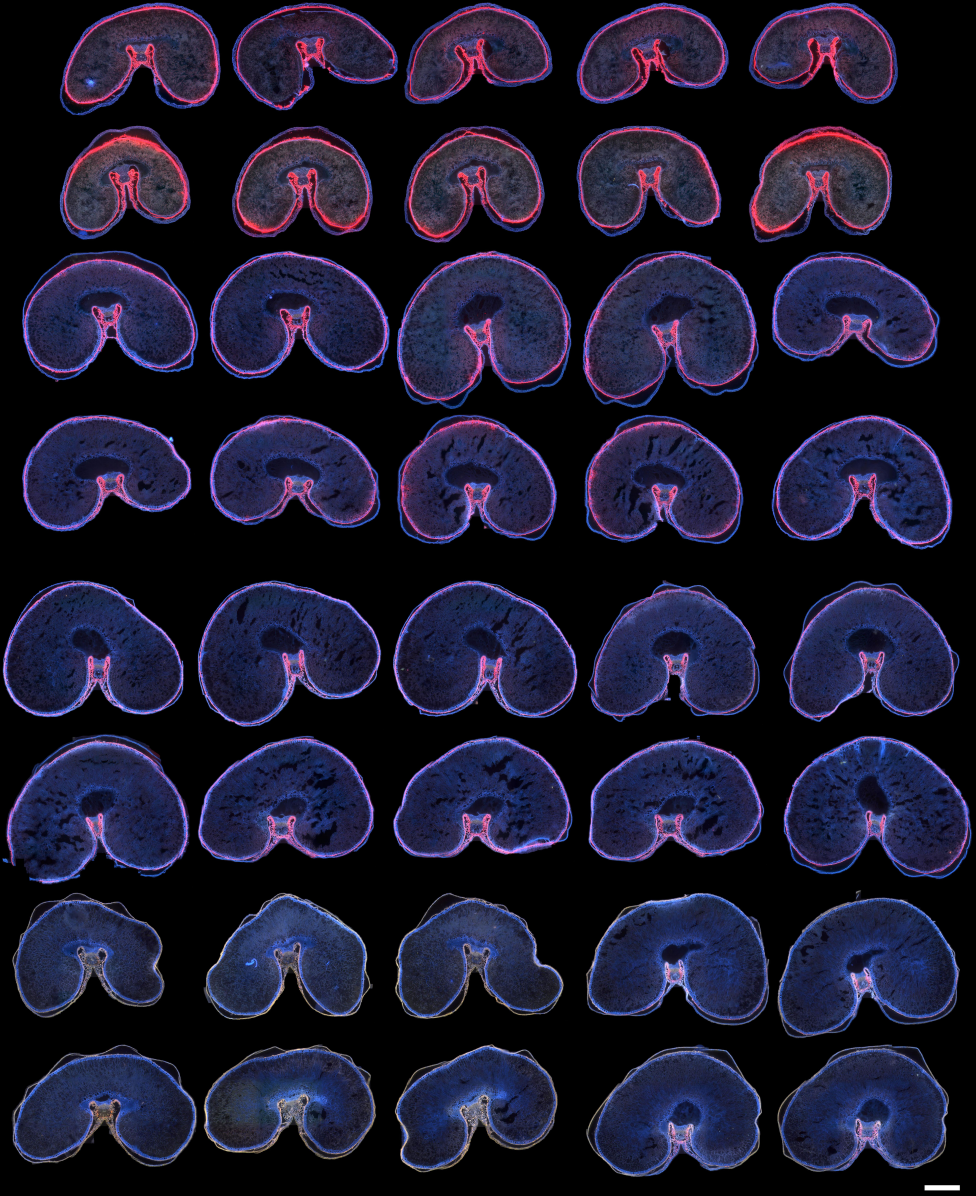
RGB composite macrofluorescence images. Sections for stages 250°DAF (lines 1 and 2), 450°DAF (lines 3 and 4), 650°DAF (lines 5 and 6), and 850°DAF (lines 7 and 8). Color intensities can be compared. Scale bar represents 1 mm.

The RGB composite macrofluorescence images shown in **Figure 3** were used to provide an overview of the whole series of images acquired for the four developmental stages.

The representation reveals that the grain size increased between 250°DAF and 650°DAF and then slightly decreased at stage 850°DAF since measured section areas were 4.64 ± 0.22, 6.56 ± 0.57, 7.11 ± 0.37, and 6.11 ± 0.46 mm^2^ for stages 250°DAF, 450°DAF, 650°DAF, and 850°DAF, respectively. The outer pericarp was often detached from the rest of the grain because of developmentally programmed cell death targeting the mesocarp (Domínguez and Cejudo, 2014).

**Figure 4:**
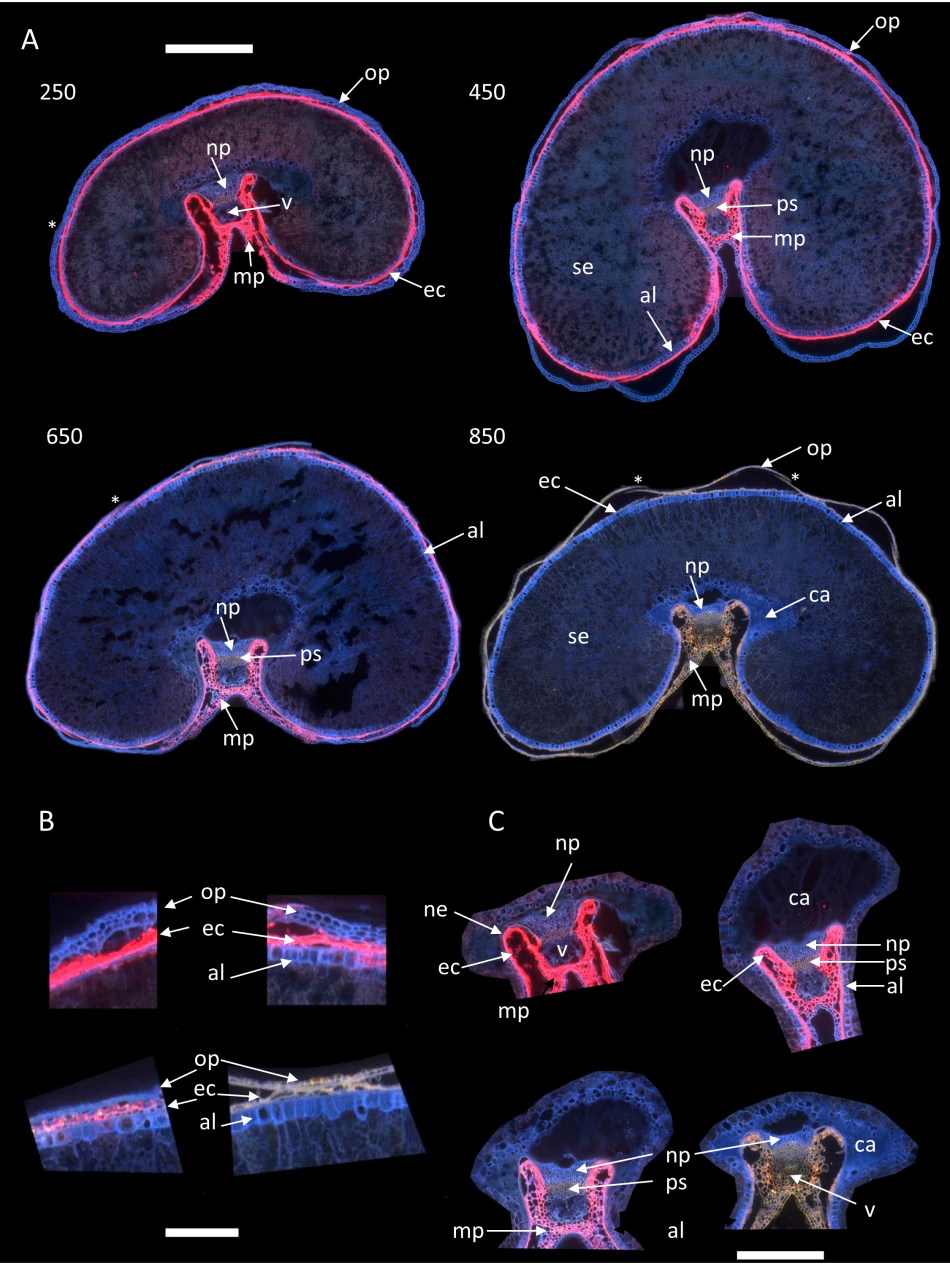
RGB composite macrofluorescence images. Examples of images for stages 250°DAF, 450°DAF, 650°DAF, and 850°DAF. **(A)** entire sections, scale bar = 1 mm; **(B)** dorsal region, scale bar = 250 µm; **(C)** crease region, scale bar = 750 µm. Color intensities can be compared. Asterisks in panel A show stitching artefacts. Mp, mesocarp; op, outer pericarp; ec, endocarp; al, aleurone; se, starchy endosperm; np, nucellar projection; v, vascular tissue; ca, apoplastic cavity; ps, pigmented strand.

Concerning fluorescence, no major differences between images of grains within the same stage were noticed. Therefore, for the rest of the manuscript, the results will be illustrated by an image per developmental stage, as in **Figure 4**. The whole series will be shown in the **Supplementary Material**. Magnified images on two regions, the dorsal and crease regions, were added to better visualize the fluorescence of tissues.

Some default in image stitching can be seen in some large images (**Figure 4A**). They concerned only a small part of the sections, did not change the fluorescence behavior, and were therefore ignored in the following.

Three dominant fluorescence colors are highlighted in the RGB composite macrofluorescence images (**Figures 3**, **4**): pink-red autofluorescence, blue autofluorescence, and orange-brown autofluorescence. The pink-red autofluorescence was mainly found in the endocarp, strong at 250°DAF, then decreasing for stages 450°DAF and 650°DAF, and not observed at stage 850°DAF. Blue fluorescence was observed in the outer pericarp at stages 250°DAF, 450°DAF, and 650°DAF; at 850°DAF, it turned to yellow-brown. The aleurone layer fluorescence was always blue, but its cells were visible from stage 450°DAF; at 250°DAF, they were not fully differentiated. Looking at the crease magnified images (**Figure 4C**), the fluorescence of the nucellar projection and the gel in the apoplastic cavity was always blue, while that of the pigment strand was always brown. The vascular bundles’ fluorescence varied according to the stages, with more blue fluorescence from stages 250°DAF to 650°DAF and brown fluorescence at stage 850°DAF. In the endosperm, a diffuse blue/red fluorescence was observed at stages 250°DAF and 450°DAF and mainly blue at stage 650°DAF. At stage 850°DAF, the endosperm cell walls were clearly visible.

Examination of the series of images alone already attested to changes in fluorescence properties during development. The objective of the following multispectral analysis is to quantify these differences and reveal more precisely the tissue variation by considering the 11 fluorescence intensities measured in the pseudo-spectra and not only the visual average fluorescence behavior.

### 3.3 Large PCA of multispectral images revealed new levels of fluorescence variability

A large PCA was performed on all the pixels of the regions of interest of the 40 images to explore signal variation on all grain sections without preconceived ideas. Five components describing 99.87% of the total variance were particularly interesting and will be described here by examining loadings and score images.

#### 3.3.1 Large principal component analysis: loadings

**Figure 5:**
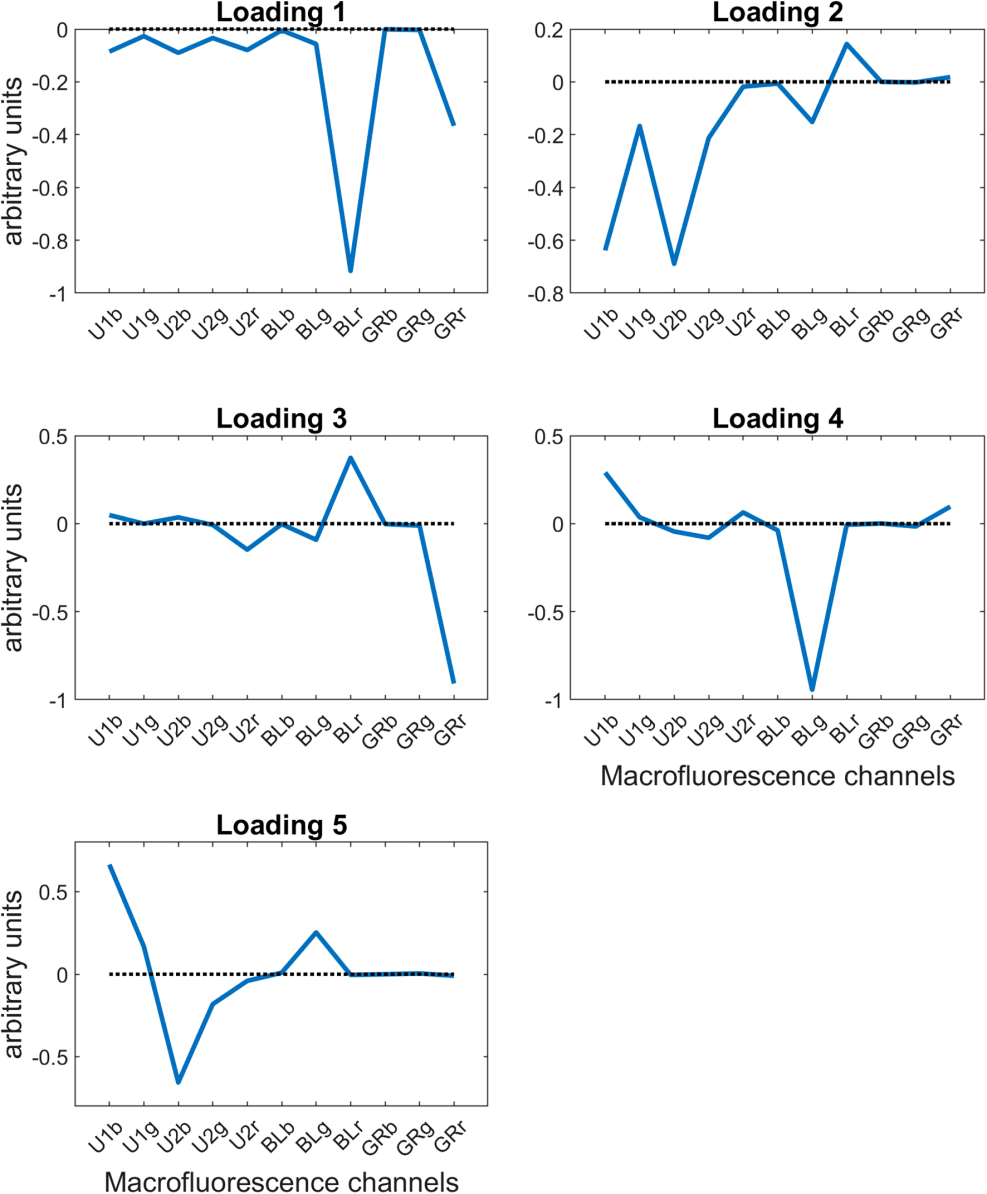
Large PCA of the series of 40 grain multispectral images: five first loadings. PCA, principal component analysis.

The loadings are shown in **Figure 5**; they express the main variations in autofluorescence present in the grain sections.

Component 1 explained 82.26% of the variability. The loading values for component 1 were all negative, indicating variation in signal intensity between pixels. It revealed differences mainly observed in red fluorescence emission after Blue and Green excitations.

Component 2 explained 13.78% of the variability and described mainly variations in fluorescence after UV excitation and partly variations in Blue light-induced fluorescence.

Component 3 explained 2.02% of the total variance. Loading 3 revealed two negatively correlated fluorescent behaviors, red emission after Green excitation and red emission after Blue excitation, highlighting relative variations in the red emissions after Blue and Green excitations.

Component 4 explained 1.30% of the variability. Loading 4 describes mainly variations of green emission after Blue excitation. This fluorescence was in contrast to a small contribution for blue emission after UV1 excitation.

Component 5 explained 0.51% of the variability. Loading 5 described mainly contrasting variations of blue emissions after UV1 and UV2 excitations. There was a small contribution of green emission after Blue excitation.

As a general result, we found three types of loadings. Loadings 1 and 3 described the variations of red fluorescence emission after Blue and Green excitations. Loadings 2 and 5 described variations in fluorescence emission after UV1 and UV2 excitations. Loading 4 described almost specifically the green fluorescence emission after Blue excitation. In the following, the score images were examined in this order.

#### 3.3.2 Large principal component analysis: score images and score distributions

**Figure 6:**
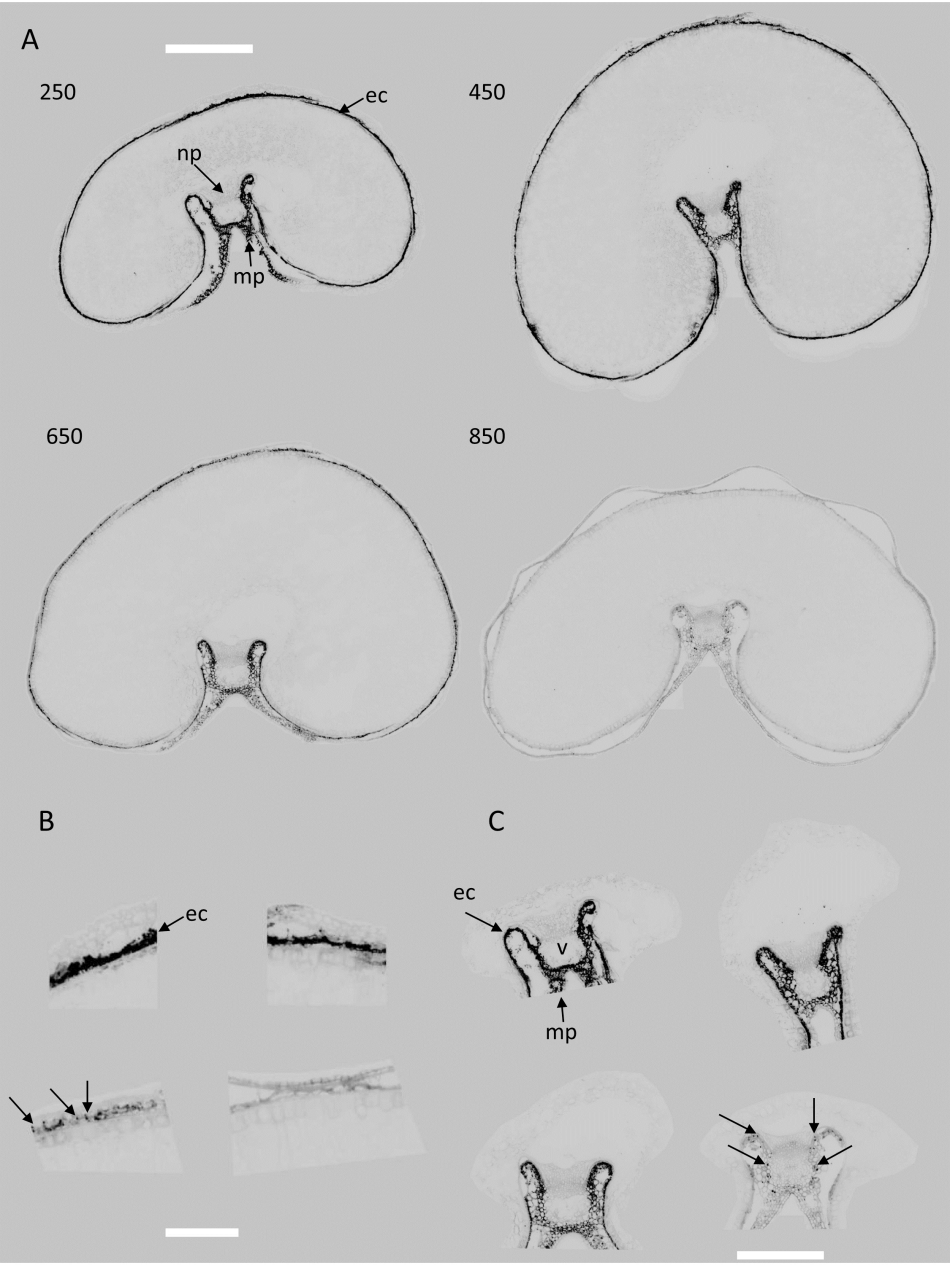
Large PCA. Component 1: examples of images and magnified images of scores for stages 250°DAF, 450°DAF, 650°DAF, and 850°DAF. **(A)** Entire sections, scale bar = 1 mm. **(B)** Dorsal region, scale bar = 250 µm. **(C)** Crease region, scale bar = 750 µm. Intensities can be compared. Unannotated arrows in panels B and C point to dots. Mp, mesocarp; ec, endocarp; np, nucellar projection; v, vascular tissue; PCA, principal component analysis.

**Figure 7:**
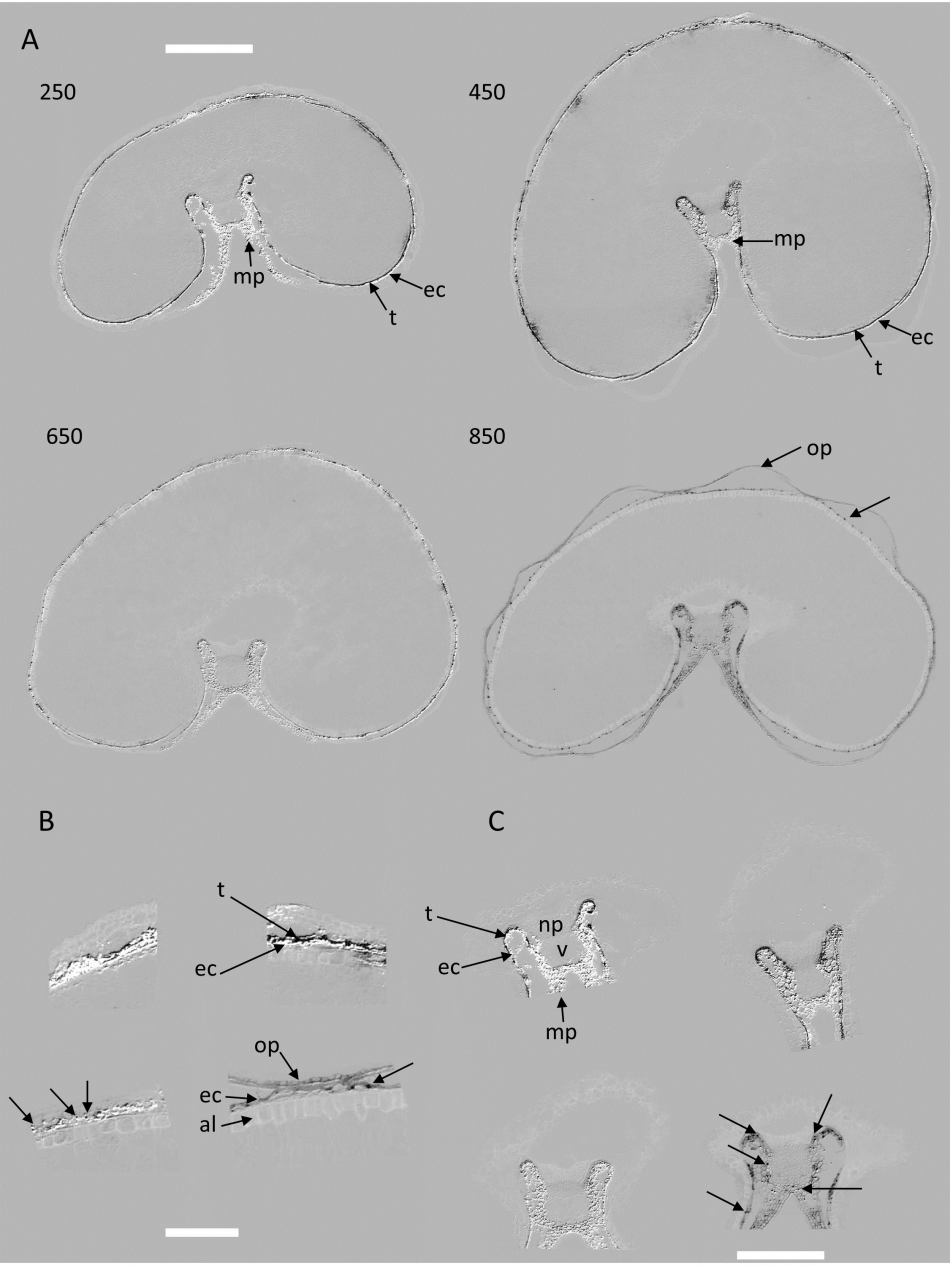
Large PCA component 3: examples of images and magnified images of scores for stages 250°DAF, 450°DAF, 650°DAF, and 850°DAF. **(A)** Entire sections, scale bar = 1 mm. **(B)** Dorsal region, scale bar = 250 µm. **(C)** Crease region, scale bar = 750 µm. Intensities can be compared. Unannotated arrows in panels **(A–C)** point to dots. Mp, mesocarp; op, outer pericarp; ec, endocarp; al, aleurone; np, nucellar projection; v, vascular tissue; t, testa; PCA, principal component analysis.

**Figure 8:**
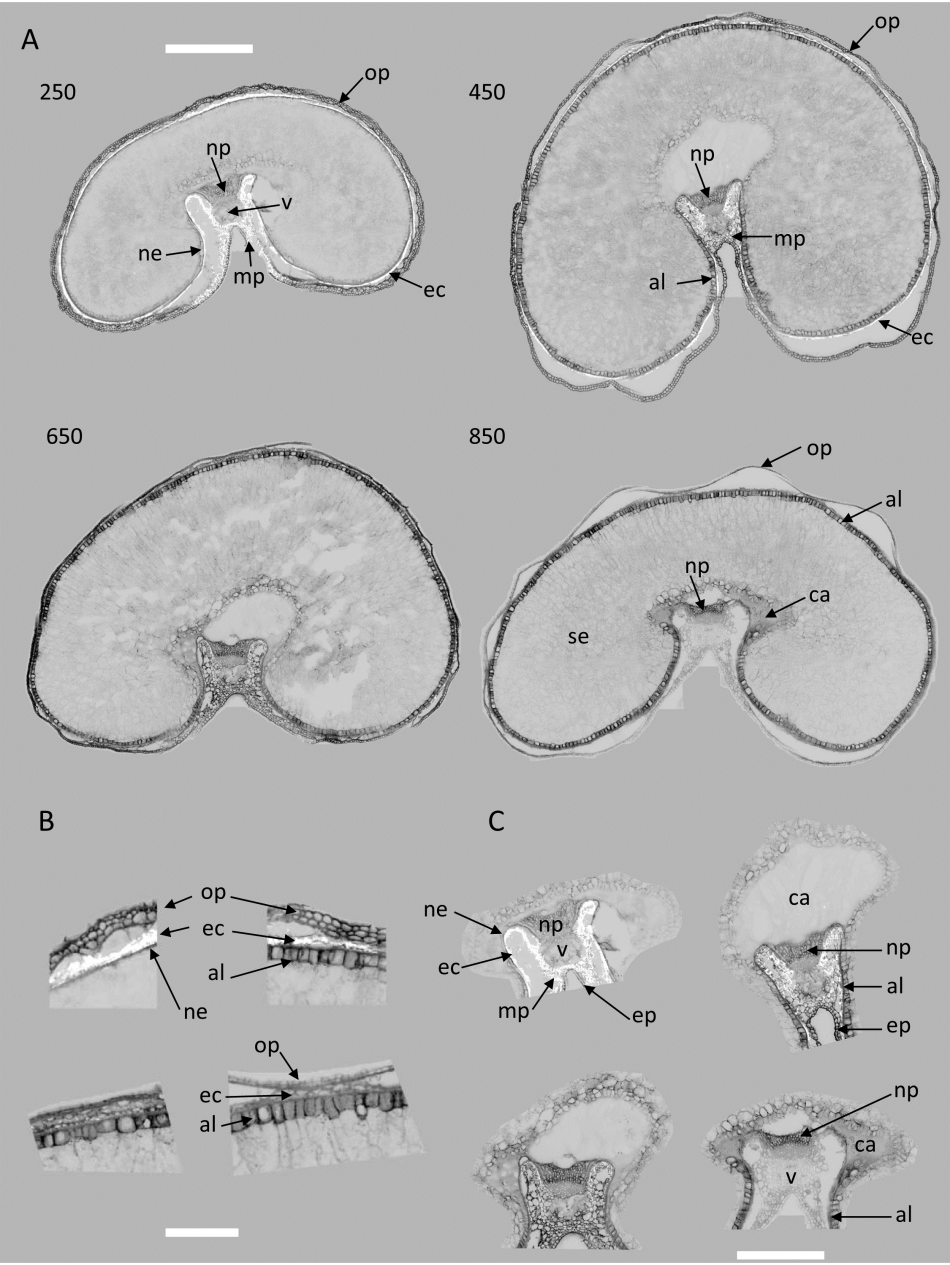
Large PCA component 2: examples of images and zoom images of scores for stages 250°DAF, 450°DAF, 650°DAF, and 850°DAF. **(A)** entire sections, scale bar = 1 mm; **(B)** dorsal region, scale bar = 250 µm; **(C)** crease region, scale bar = 750 µm. Intensities can be compared. Ep: epicarp; mp, mesocarp; op, outer pericarp; ec, endocarp; ne, nucellar epidermis; al, aleurone; se, starchy endosperm; np, nucellar projection; v, vascular tissue; ca, apoplastic cavity; PCA, principal component analysis.

**Figure 9:**
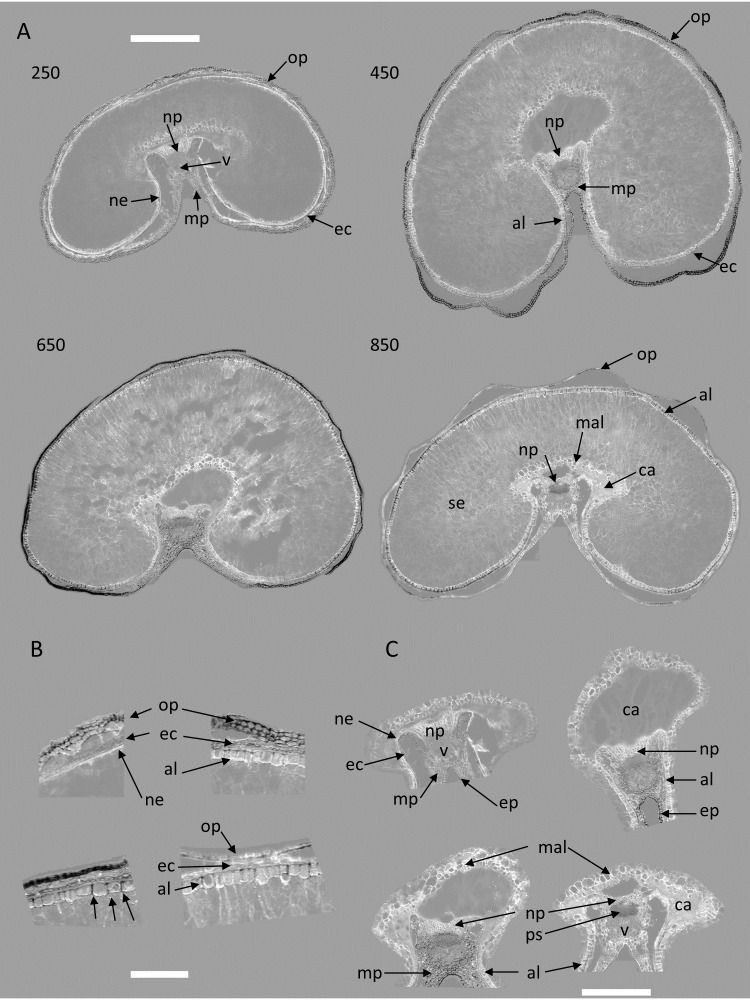
Large PCA component 5: examples of images and zoom images of scores for stages 250°DAF, 450°DAF, 650°DAF, and 850°DAF. **(A)** Entire sections, scale bar = 1 mm. **(B)** Dorsal region, scale bar = 250 µm. **(C)** Crease region, scale bar = 750 µm. Intensities can be compared. Unannotated arrows in panel B point at synclinal and anticlinal cell wall differences for aleurone. Ep: epicarp; mp, mesocarp; op, outer pericarp; ec, endocarp; ne, nucellar epidermis; al, aleurone; se, starchy endosperm; mal, modified aleurone; np, nucellar projection; v, vascular tissue; ca, apoplastic cavity; ps, pigmented strand; PCA, principal component analysis.

**Figure 10:**
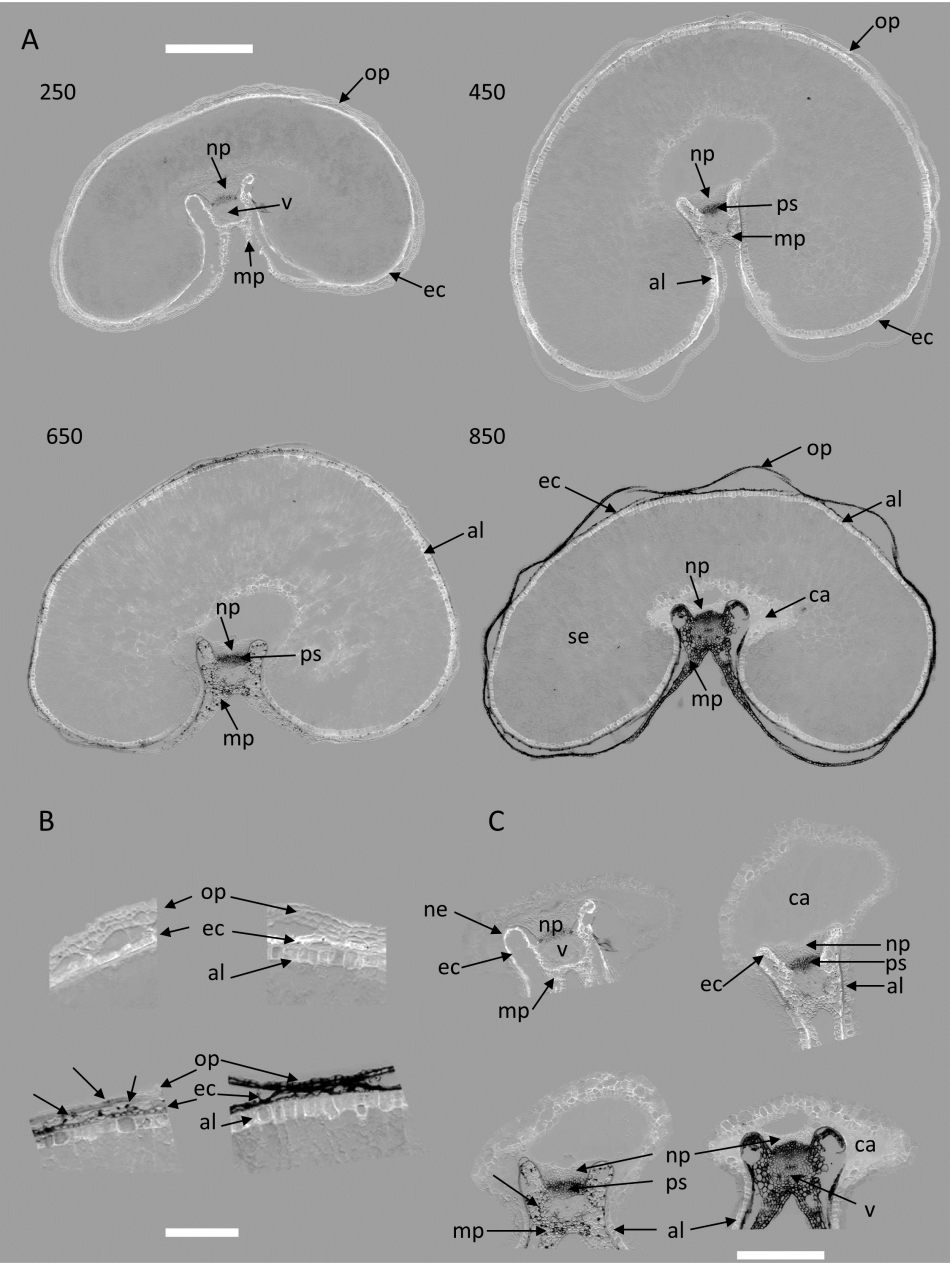
Large PCA component 4: examples of images and zoom images of scores for stages 250°DAF, 450°DAF, 650°DAF, and 850°DAF. **(A)** Entire sections, scale bar = 1 mm. **(B)** Dorsal region, scale bar = 250 µm. **(C)** Crease region, scale bar = 750 µm. Intensities can be compared. Unannotated arrows in panels B and C point to dots. Mp, mesocarp; op, outer pericarp; ec, endocarp; al, aleurone; se, starchy endosperm; np, nucellar projection; v, vascular tissue; ca, apoplastic cavity; ps, pigmented strand; PCA, principal component analysis.

Score images reveal the localization of the spectral variations described by each component. **Figures 6**–**10** illustrate the score images for a representative entire section at each developmental stage and magnified images in the dorsal and crease area of the corresponding section (the whole series can be seen in the **Supplementary Material**). Spectral characteristics, as well as tissue and subcellular localization, and knowledge of grain composition were used to assign chemical compounds to large PCA components.

In order to quantify the differences observed in the score images, score distributions were computed for the region of interest corresponding to the outer layers of the grains, therefore discarding the endosperm pixels (see regions of interest in the **Supplementary Material** supplementaryRoiOuterTissues).

##### 3.3.2.1 Large PCA components 1 and 3: red fluorescence emission after Blue and Green excitations

###### 3.3.2.1.1 Score images 1

Component 1 of the image series, which reveals the differences in red fluorescence emission, highlights a negative black signal located at the periphery of the grain (**Figure 6**). It was observed for two tissues: the endocarp and the mesocarp close to the vascular tissue.

From the images, it is clear that scores decreased and showed different patterns across development. While in the early stages the fluorescence was high and homogeneous, at 650°DAF, it was irregular and formed dots. At 850°DAF, the remaining fluorescence was weak, but small dots were revealed in various places, particularly in the crease region (**Figure 6C**, see also **Supplementary CP1**). Both the characteristics of loading 1 with red emission after Blue and Green excitations and the fact that the signal was detected in tissues known to contain chloroplasts indicated that component 1 can be attributed to chlorophyll. Indeed, chlorophyll is known to absorb a wide range of light wavelengths, emitting mainly red and far-red fluorescence (Buschmann et al., 2000; Buschmann, 2007). The decrease in signal intensity at the end of the development agrees with the fact that the chlorophyll in wheat grain is known to be degraded late in grain development and absent from dry grains. Dots observed at late stages could be due to remaining chlorophyll.

**Figure 11:**
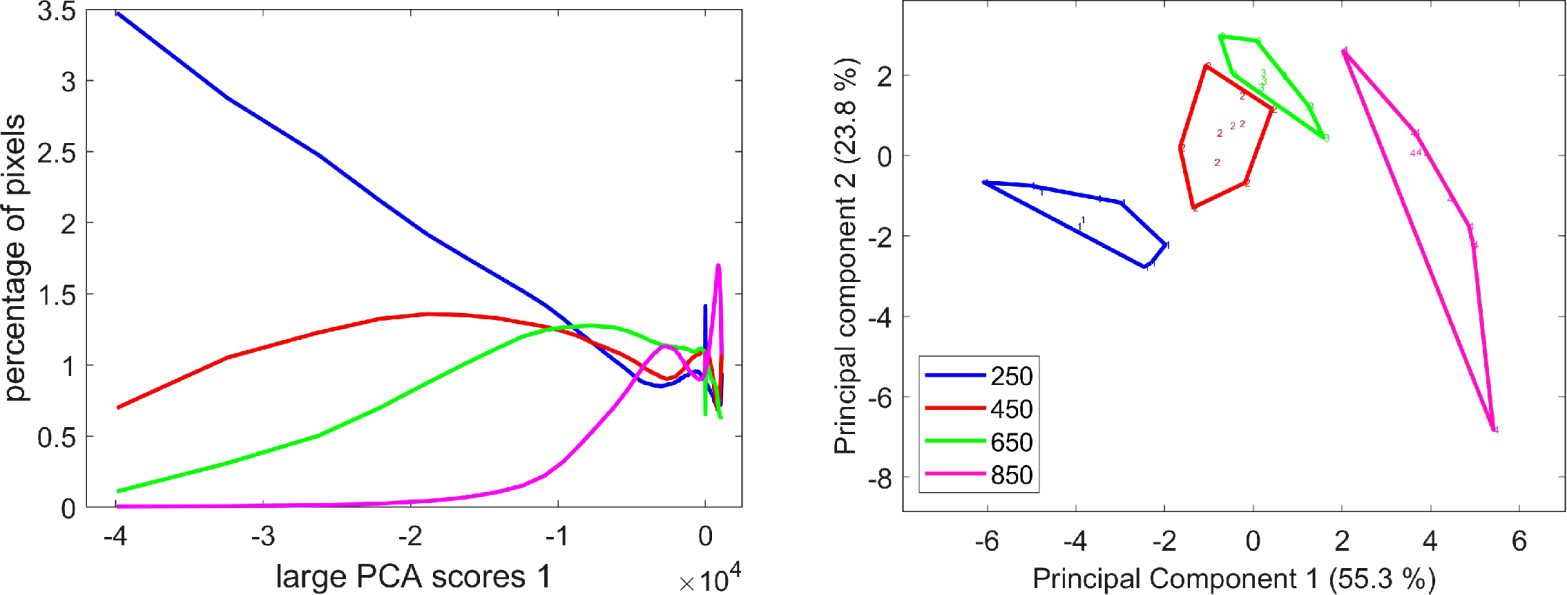
Large PCA. Component 1: average score distributions and principal component analysis of the score distributions; similarity map. Loadings can be found in the **Supplementary Material**. PCA, principal component analysis.

On average, the corresponding score distributions clearly highlight the decreasing proportion of pixels with negative scores, i.e., high red fluorescence emission, from stage 250°DAF to stage 650°DAF (**Figure 11**). Almost no pixels with large PCA score values below −1.5–10^4^ were observed for stage 850°DAF. The PCA of the score distributions allows for the comparison of the whole image series at a glance. The similarity map (**Figure 11**) demonstrates that the differences observed between the distributions depend on the stage of development. It clearly attests to the relative evolution of red fluorescence emission from stages 250°DAF to 850°DAF, showing the variability both in the same stage and between each stage.

The interpretation tools of large PCA, i.e., score images and score distributions, proved to be relevant for comparing the series of multispectral images, detecting differences between developmental stages, and indicating the tissue origin of these differences for component 1. For the other components, we propose to start with the analysis of the score distribution to obtain an immediate overview of the variability observed in the image series. We will then move on to the spatial interpretation by examining representative score images.

###### 3.3.2.1.2 Score image 3

Whereas component 1 described the general variations in the intensity of the red emissions after Blue and Green excitations, component 3 describes the relative variations between these two emissions. For some pixels, the red emission after Blue excitation was higher than the red emission after Green excitation.

**Figure 12:**
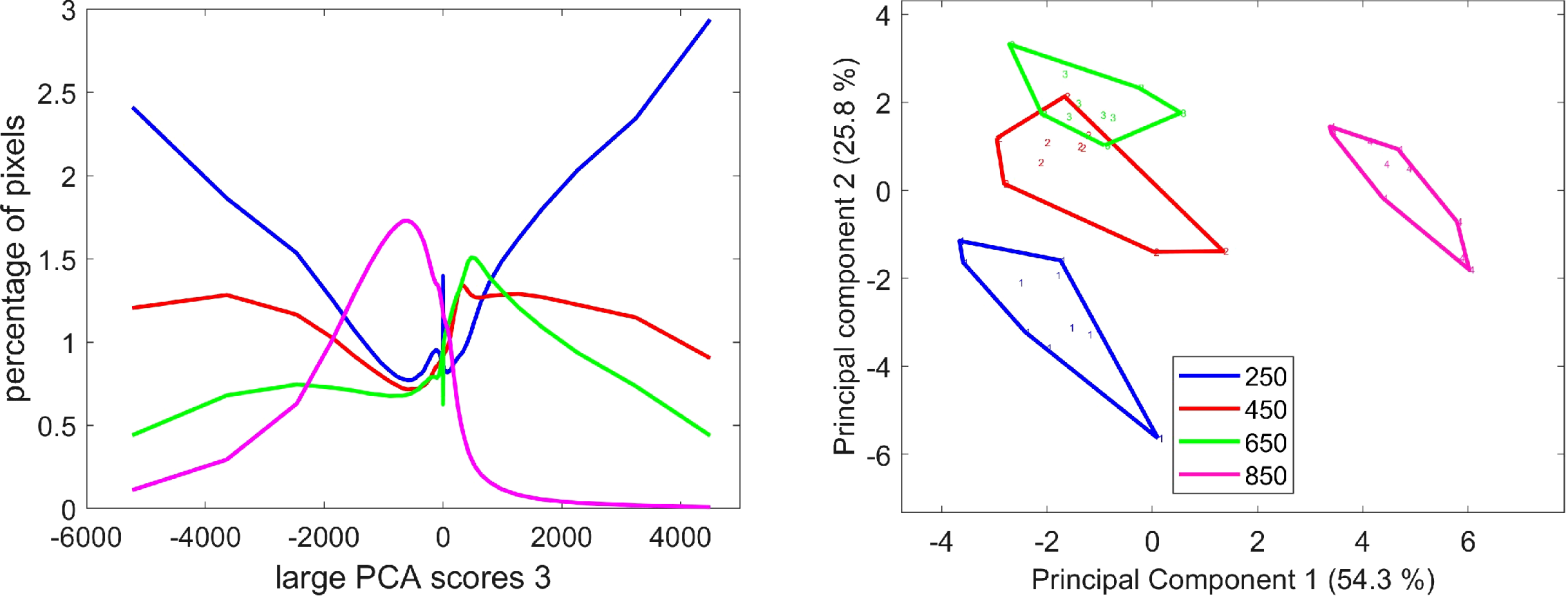
Large PCA component 3: average score distributions and principal component analysis of the score distributions: similarity map. Loadings can be found in the **Supplementary Material**. PCA, principal component analysis.

The average score distributions and their PCA (**Figure 12**) show that these variations were also related to the developmental stage. The majority of pixels with strong negative and positive scores were found in the 250°DAF stage. Their proportions decreased for stages 450°DAF and 650°DAF. The shape of the distribution was completely different for stage 850°DAF, for which the highest proportion of pixels was found for pixels with low negative scores. The PCA of the score distributions attests to the strong differences between stage 850°DAF and the other three stages. The continuous variations between stages 250°DAF and 650°DAF were observed on component 2 of the score distributions.

The example score images in **Figure 7** allow for locating the fluorescence variations that occurred. Two types of score images were observed. Stages 250°DAF to 650°DAF followed similar patterns with black (negative scores) and white (positive scores) pixels in the region, including the testa, nucellar epidermis, and endocarp, and in the crease mesocarp. At 850°DAF, mainly light dark pixels (low negative scores) were observed for the same tissue and also in the outer pericarp.

Compared to large PCA component 1, between 250°DAF and 650°DAF, the signal variations were located in the same tissues (**Figure 6** compared to **Figure 7**), which was attributed to chlorophyll. In the crease region, the signal was detected in the loops flanking the nucellar projection with an interruption in the pigment strand region (**Figure 7C**). Two tissues follow this spatial arrangement in the wheat grain, the nucellar epidermis and the testa, which cannot be clearly distinguished by macroscopy. In the lobe area, white pixels with higher red emission after Blue excitation formed a white line visible below a black line of pixels. In the dorsal region, the signal was noisier; it seemed that several black lines of pixels, i.e., with a higher relative red emission after Green excitation, were visible, especially for grains at 450°DAF (**Supplementary CP3**). It should be noted that in the 850°DAF stage, the outer pericarp shows a continuous dark signal, unlike in the other three stages. The endocarp shows clearly marked dots that can also be distinguished at stage 650°DAF.

Possible molecular compounds for the part of component 3 with negative scores could be flavonoids. Indeed, some forms of flavonoids were reported to emit red fluorescence after Green excitation (Garcia-Plazaola et al., 2015). Flavonoids are present in chloroplasts; some, such as tricin, were detected incorporated in lignin of wheat straw and rice grain outer tissues (Lam et al., 2021; Poulev et al., 2018) and others in cuticles in other species (naringenin in tomato (Adato et al., 2009). Moreover, both wheat testa and nucellar epidermis are covered by a cuticle.

##### 3.3.2.2 Large PCA components 2 and 5: blue emission after UV excitation

###### 3.3.2.2.1 Score image 2

**Figure 13:**
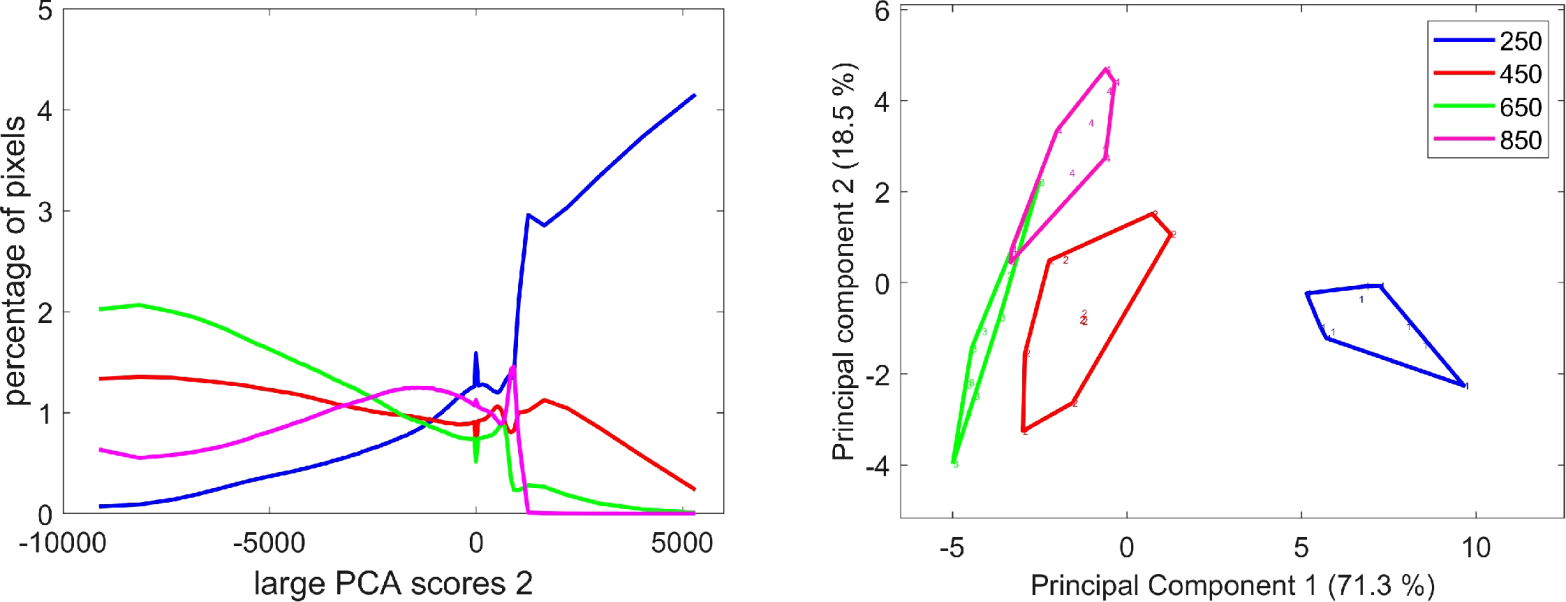
Large PCA component 2: average score distributions and principal component analysis of the score distributions; similarity map. Loadings can be found in the **Supplementary Material**. PCA, principal component analysis.

The analysis of the score distributions of large PCA component 2 also shows that the fluorescence properties described by this component depended on the stage of development (**Figure 13**). In large PCA component 2, the negative scores (see loading in **Figure 5**) corresponded to pixels that emitted a high blue fluorescence after UV (UV1 and UV2) excitations. The positive scores were associated with variations in red emission after Blue excitation. They were observed mainly for the 250°DAF stage and to a much lesser extent for the 450°DAF stage (**Figure 13**). The amount of UV fluorescence increased between 250°DAF and 650°DAF. The score distributions measured that the UV fluorescence intensity was lower at stage 850°DAF compared to stages 450°DAF and 650°DAF.

Looking at the example score images in **Figure 8**, blue fluorescence after UV excitations (black pixels) was detected in the cell walls of the outer pericarp and nucellar projection at all stages; at stages 450°DAF, 650°DAF, and 850°DAF in the endocarp and aleurone layer; and at 850°DAF in the cell walls of the starchy endosperm and in the gel present in the apoplastic cavity. The increase of fluorescence intensity after UV excitation can be seen between 250°DAF and 650°DAF in the pericarp, aleurone, and nucellar epidermis. Between 650°DAF and 850°DAF, the fluorescence intensity decreased in the outer pericarp and the crease mesocarp.

Blue fluorescence emission after UV excitations is generally assigned to phenolics, including hydroxycinnamic acids and lignin (Garcia-Plazaola et al., 2015; Donaldson, 2020). Hydroxycinnamic acids are mainly excited under UV excitation and emit an intense blue fluorescence (Fulcher et al., 1972; Harris and Hartley, 1976), while lignin is excited under both UV and visible light and emit blue, green, and red fluorescence (Djikanović et al., 2007; Donaldson, 2013, 2020). The cell walls of cereal grains contain ferulic and *p*-coumaric acid, two hydroxycinnamic acids very similar in structure, which are linked to hemicellulose (xylan) and lignin (Chateigner-Boutin and Saulnier, 2022).

The differences in the cell wall composition, linkages, and properties of phenolics were reported between wheat grain tissues and developmental stages (Barron et al., 2007; Chateigner-Boutin et al., 2018). In particular, the cell walls of the nucellar epidermis and those of the aleurone are known to contain relatively large amounts of ferulic acid, a smaller amount of *p*-coumaric acid, and traces of lignin. Ferulic acid linked to xylan has also been reported in the gel found in the apoplastic cavity (Chateigner-Boutin et al., 2021). Pericarp cell walls are known to contain both hydroxycinnamic acids and lignin (Chateigner-Boutin et al., 2018). Hence, both hydroxycinnamic acids and lignin could contribute to component 2. The decrease in fluorescence intensity measured in the pericarps at stage 850°DAF compared to earlier stages could be due to changes in the environment of these compounds, such as changes in macromolecule interactions or the drop in water content occurring at the end of grain development (Chateigner-Boutin et al., 2018).

###### 3.3.2.2.2 Score image 5

**Figure 14:**
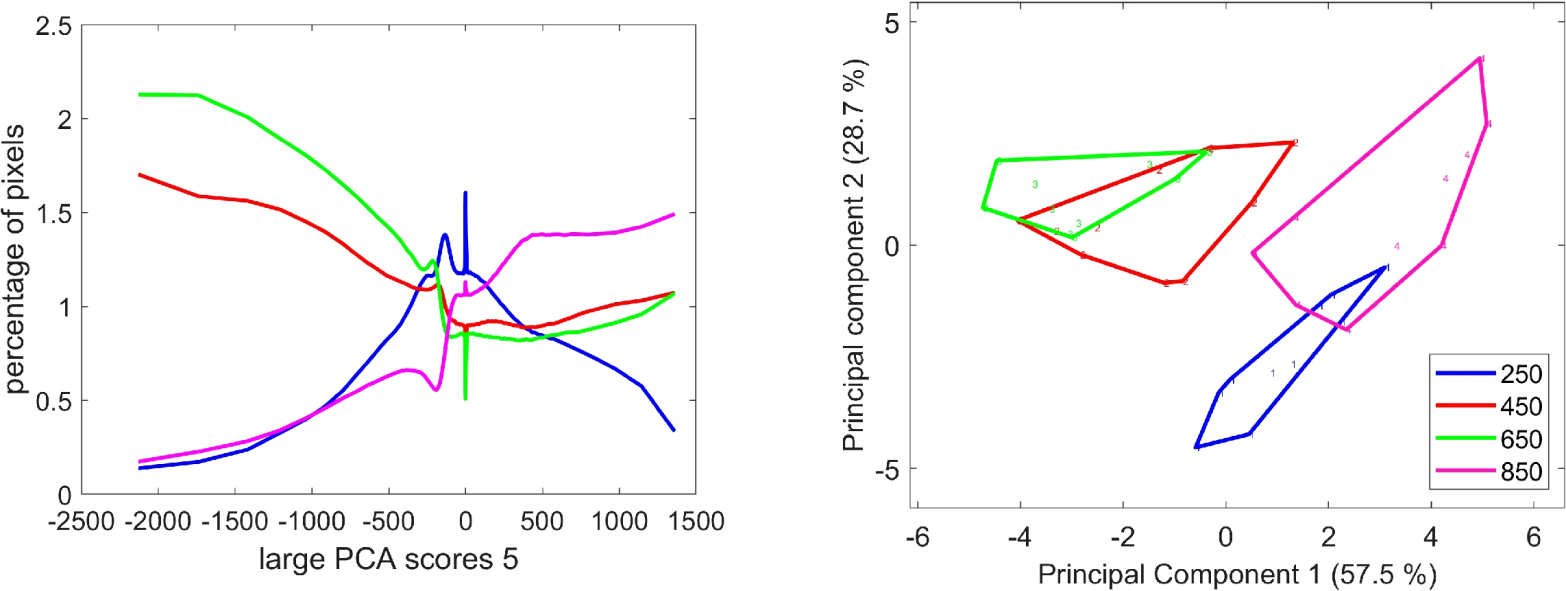
Large PCA component 5: average score distributions and principal component analysis of the score distributions; similarity map. Loadings can be found in the **Supplementary Material**. PCA, principal component analysis.

Large PCA component 2 described mainly UV fluorescence intensity, and component 5 described relative fluorescence emissions after UV1 and UV2 excitations. The positive score values correspond to higher blue emission after excitation in UV1, and the negative score values represent a higher blue emission after excitation in UV2. The score distribution analysis (**Figure 14**) shows that these relative variations depended on the developmental stages. More pixels with high relative UV2 excitation were observed for stages 450°DAF and 650°DAF, while the highest percentage of pixels with a high relative UV1 excitation was found at stage 850°DAF. Stage 250°DAF was distinguished by less contrasting fluorescence behavior than the other three stages.

The score images (**Figure 9**) show that black pixels with negative scores, i.e., relatively high UV2 fluorescence, were mainly detected in the outer tissues of the grains, more precisely in the cell walls of the outer pericarp at stages 450°DAF and 650°DAF, and of the aleurone at stages 650°DAF and 850°DAF. In the pericarp, the UV2 relative fluorescence increased from stages 250°DAF to 650°DAF. It almost fully disappeared at stage 850°DAF, for which most pixels in the pericarp were white, i.e., showing a higher UV1 fluorescence. The endocarp fluorescence did not vary much from stages 250°DAF to 650°DAF, with a slightly higher UV2 relative fluorescence. Again, at stage 850°DAF, the endocarp showed a higher UV1 fluorescence.

In the aleurone layer, a variability was observed between different cell walls. Anticlinal walls mostly appeared black at all stages from 450°DAF, while the inner synclinal cell wall next to the starchy endosperm tissue appeared mostly white, probably reflecting a polar variability in cell wall composition between these two types of cell walls. Polar variability in cell wall composition has already been reported (Dauphin et al., 2022). Moreover, a regional variability was evidenced since more negative pixels were found in the cell walls of the aleurone in the lobe and dorsal regions compared to the crease region, and the polar variability was not observed in the cell walls of the modified aleurone.

Component 5 may be attributed to phenolic acids and lignin, and relative UV1 and UV2 fluorescence variations may represent different forms of phenolics. One possibility could be relative differences in *p*-coumaric versus ferulic acid composition or differences due to linkages between hydroxycinnamic acids and cell wall polymers.

##### 3.3.2.3 Large principal component 4: emission in green after Blue excitation

**Figure 15:**
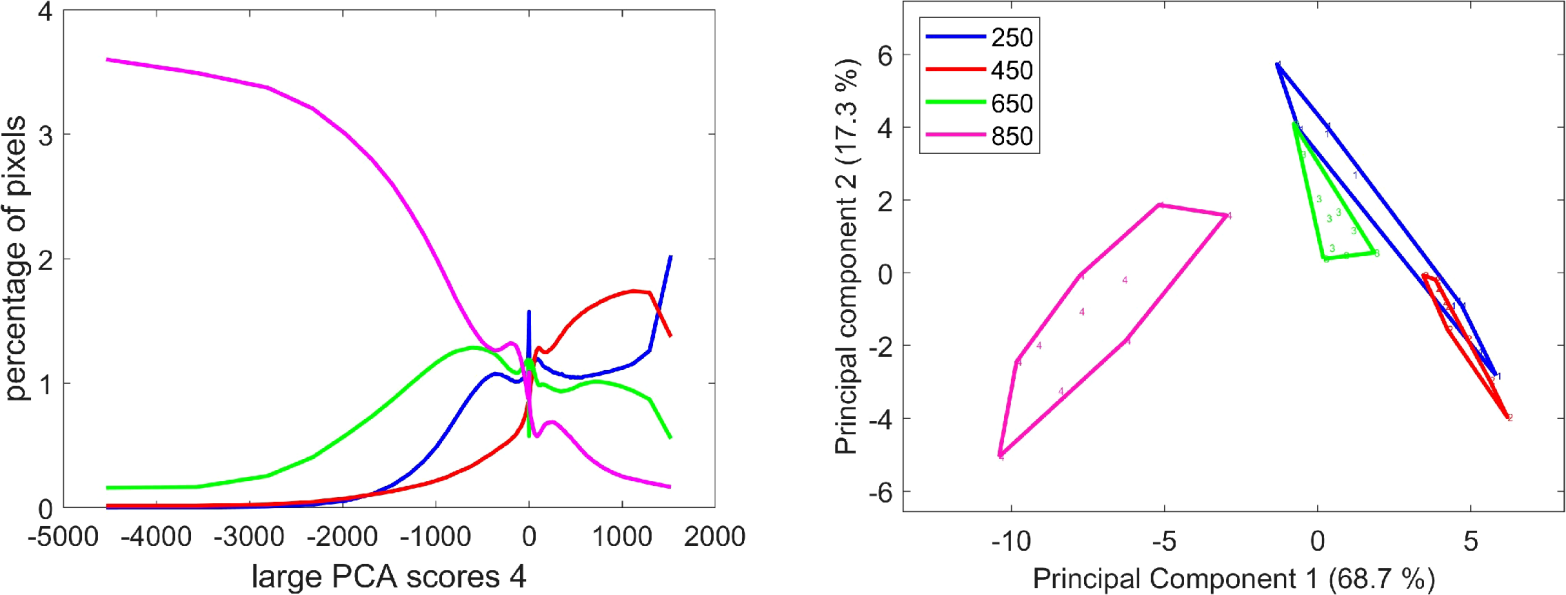
Large PCA component 4: average score distributions and principal component analysis of the score distributions; similarity map. Loadings can be found in the **Supplementary Material**. PCA, principal component analysis.

Large PCA component 4 mainly described the variation in green emission after Blue excitation, as shown by loading 4 corresponding to negative scores. The average score distributions show that these pixels were mostly found at stage 850°DAF. The PCA of the score distributions highlights a clear difference between this stage and the other three. The shape of the average score distributions shows an increase of dark pixels from stages 450°DAF to 850°DAF, together with a decrease of white pixels with positive scores. These latter pixels are characterized by a higher blue emission after UV1 excitation. The distribution of scores at stage 250°DAF did not follow the same trend. The corresponding group of images of the grain section was not clearly distinguished on the similarity map (**Figure 15**).

Looking at the whole series of score images in **Supplementary CP4**, at stage 250°DAF, two fluorescence behaviors were observed. In the second row of the whole section panel at 250°DAF, the endosperm showed negative scores, i.e., green emission after Blue excitation, which was barely observed for the first row. The sections shown on the first row came from three grains and those of the second row from two grains, indicating a clear grain effect. In addition, this fluorescence was not observed in cell walls but inside cells. This green fluorescence could be assigned to families of compounds as described in Garcia-Plazaola et al. (2015), like riboflavin (Batifoulier et al., 2006), flavonoids and carotenoids (Adom et al., 2005), or terpenoids (Shewry and Hey, 2015), which can be found in the wheat grain. As our main interest was on outer layers, these specific variations inside the endosperm will not be further commented on.

In the examples of score images in **Figure 10**, black pixels with negative scores were mainly detected in the outer tissues of the grains at stage 850°DAF, more precisely in the cell walls of the pericarp, outer pericarp, and endocarp. Light dark pixels with low negative scores were found at stage 650°DAF with a heterogeneous spatial distribution. The positive score values are characterized by a relative Blue emission after UV1 excitation observed in the cell walls of the aleurone layer and the nucellar projection for all stages and in the pericarp at the earliest stages of 250°DAF and 450°DAF.

The analysis of the spatio-temporal variability in entire grain sections revealed the absence of black pixels with negative scores in most cell walls of grains at 250°DAF and 450°DAF. There were exceptions, such as the pigment strand region and some cell walls of the vascular tissues. At 450°DAF, a closer observation, especially in zoom images (see **Supplementary CP4** and **Figure 13B**, dorsal zoom), revealed that black dots with negative scores can be found in the endocarp. At 650°DAF, more dots were detected, and they were also present in the outer pericarp and ventral mesocarp (**Figure 10**). The dots in the endocarp seemed to be localized at the junction of endocarp cells and could represent cell wall microdomains with a specific composition. At 850°DAF, the cell walls of all outer tissues, outer pericarp, mesocarp, endocarp, testa, and vascular bundle showed high negative scores for component 4. In contrast, the cell walls of the starchy endosperm, aleurone, and nucellar projection were characterized by white pixels with positive scores, i.e., a higher blue emission after UV1 excitation.

Emission in green after excitation in blue has been reported for several compounds present in wheat, such as flavonoids, terpenoids, carotenoids, alkylresorcinols, and hydroxycinnamic acid-containing lignin (Donaldson, 2013, 2020; Verdeal and Laurenz, 1977; Agati et al., 2009). At 850°DAF, most pixels with negative scores are located in cell walls of outer layers, which is consistent with an attribution to lignin.

In wheat grain, the presence of lignin has been reported in the cell walls of the mature pericarp and measured by chemistry, with an amount of approximately 1%–2% in the outer pericarp. It has been detected in the outer layers of developing grains at stages as early as 250°DAF–450°DAF, and developmental changes in lignin structure were indirectly evidenced (Chateigner-Boutin et al., 2018). Changes in lignin structure and density according to developmental stages may affect its fluorescence properties, and these changes may correspond to the negative scores of component 4. We attributed the negative signal of component 4 to lignin at the latest stage.

Moreover, several articles reported the presence of lignin in the pigment strand (Zee and O’Brien, 1970; Lingle and Chevalier, 1985), another element in favor of the negative signal of component 4 being due to lignin.

Component 4 formed localized patches in the outer layers and had a highly heterogeneous distribution until 850°DAF, where it is more homogeneously distributed. Interestingly, it was proposed that numerous initiations of lignification may occur in young tissues of the Poaceae species, which could form small pools of lignin. These pools would subsequently connect as lignification increases (Hatfield et al., 2017). The patches of component 4 may represent these pools of lignin.

The dot signal in the endocarp seemed organized, possibly at the junctions between endocarp cells. Some dots in the mesocarp were remarkably revealed by both signals of components 3 and 4 (**Figures 7**, **10**).

## 4 Conclusion

This work demonstrates how multispectral autofluorescence analysis of plant organ sections combined with large PCA is a powerful tool to study the relationship between tissue chemical composition, its variability, and organ internal structure at the macroscopic scale.

The macroscopic scale is important to study a characteristic and its variability in entire sections of an organ. Conducted on relatively thick sections without prior fixation, embedding, or labeling to reveal native autofluorescence signals, the macroscope scale covers the entire section and allows the development of a statistical approach to imaging. There are limitations since, due to resolution limits, it does not allow the distinction of different thin tissues and structures such as the testa, the nucellar epidermis, and their cuticles, for instance, for wheat grains. The macroscopic scale should be used as a preliminary step to microscopic studies, which provide information with a higher spatial resolution but in a limited field of view, i.e., a few cells and even a cell wall or an organelle.

In the present work, variations in the chemical composition of the outer tissues of the wheat grains were studied through their autofluorescence properties. Fluorescence is known to depend on the chemical environment, to be influenced by other neighboring compounds (quenching phenomena, for instance) and by linkages and interactions between compounds. Nevertheless, our results based on the statistical analysis of several grains for the same stage of development evidence that the fluorescence signature does depend on the grain tissues and their stage of development. Based on the literature and autofluorescence properties, we suggest possible interpretations including chlorophyll, flavonoids, phenolics, and lignin fluorescence.

The 40 large multispectral images of wheat grain sections could be compared using PCA adapted for large image series. In this way, we have successfully highlighted new information. Five principal components were interpreted, revealing new levels of variability and allowing quantification and comparison between images.

The five principal components were attributed to chemical compounds or families of compounds based on their spectral features and localization. A particularly high spatial variability was observed in the crease region composed of multiple tissues and structures (chlorophyll-containing mesocarp and endocarp, vascular tissues, aleurone layer, nucellar epidermis and nucellar projection, pigmented strand, and cuticles). Developmental changes in fluorescence behaviors were highlighted. We were able to measure changes in the specific fluorescence of chlorophyll during grain development, as well as the sharp change in the fluorescence of the pericarp, linked to its lignification at the latest stage.

Among the new information revealed by this approach, large PCA highlighted local specificities. An atypical distribution of component 4 attributed to lignin was highlighted, with heterogeneity appearing in the grain outer layers with a patchy distribution, becoming homogeneous in the outer layers at the last stage of development. Moreover, dots and polar distributions were revealed. While some dots could correspond to plastids in the endocarp and the crease mesocarp, other local heterogeneities were mapped on cell walls. Dots were mapped at the junctions of endocarp cells for component 4, attributed to lignin, and dots and polar distributions were found (synclinal/anticlinal cell walls) for component 5, attributed to phenolics. These local heterogeneities could be due to local differences in chemical composition and/or cross-linkages in cell walls. The contribution of other factors, such as differences in cell wall thickness or the combination of two cell walls of different composition from neighboring cells, cannot be ruled out.

Multiscale and multimodal, e.g., fluorescence, Raman, and infrared spectroscopies, approaches applied to regions of high fluorescence variability could help determine tissue composition and interpret their fluorescence signals.

As a general conclusion, our analysis on a macroscopic scale has enabled us to observe variability on the scale of entire cross-sections in equatorial position and across four stages of development. It would be interesting then to extend this study to different positions in the grain, to different wheat genotypes and different growth conditions (e.g., normal versus stressed conditions), to explore how this variability can relate to differences in tissue composition and growth parameters. Hence, this method could help understand how local cell wall variability could impact wheat grain shape or identify wheat genotypes tolerant to climate stress.

## Data Availability

The raw data supporting the conclusions of this article will be made available by the authors, without undue reservation.
