# Supplementary material for: Exploring the structural variability in developing wheat grains using autofluorescence multispectral imaging at the macroscopic scale
Source: Front Plant Sci. 2025 Jun 19;16:1580426. doi: 10.3389/fpls.2025.1580426 (PMC12222247; doi:10.3389/fpls.2025.1580426)
Supplement: Supplementary file 1 [file DataSheet1.zip › SupplementaryData/Supplementary Table 1.docx]

**Supplementary Table 1.** Characteristics of the four fluorescence filter cubes, acquisition time gain, lamp power and a posteriori multiplicative factor

| Filter code | Excitation filter  Band pass (nm) | Dichroic mirror (nm) | Emission filter  long pass (nm) | Acquisition time  (ms) | Gain | Lamp power (%) | Multiplicative factor |
| --- | --- | --- | --- | --- | --- | --- | --- |
| U1 | 327-353 | > 380 | > 364 | 1000 | 2 | 100 | 1 |
| U2 | 325-375 | > 400 | > 420 | 300 | 1 | 50 | 1 |
| BL | 460-490 | > 500 | > 515 | 250 | 1 | 50 | 5 |
| GR | 510-560 | > 565 | > 590 | 200 | 1 | 50 | 5 |
