# Supplementary material for: Exploring the structural variability in developing wheat grains using autofluorescence multispectral imaging at the macroscopic scale
Source: Front Plant Sci. 2025 Jun 19;16:1580426. doi: 10.3389/fpls.2025.1580426 (PMC12222247; doi:10.3389/fpls.2025.1580426)
Supplement: Supplementary file 1 [file DataSheet1.zip › SupplementaryData/SupplementaryCP3.docx]

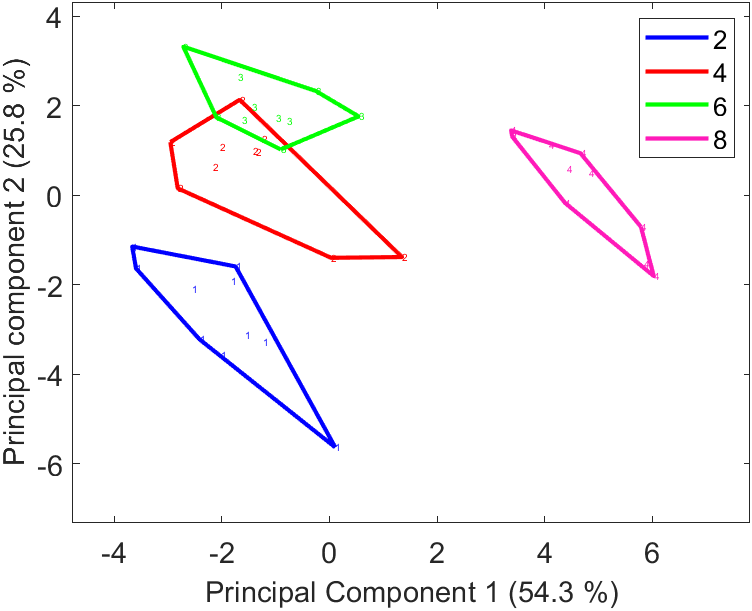

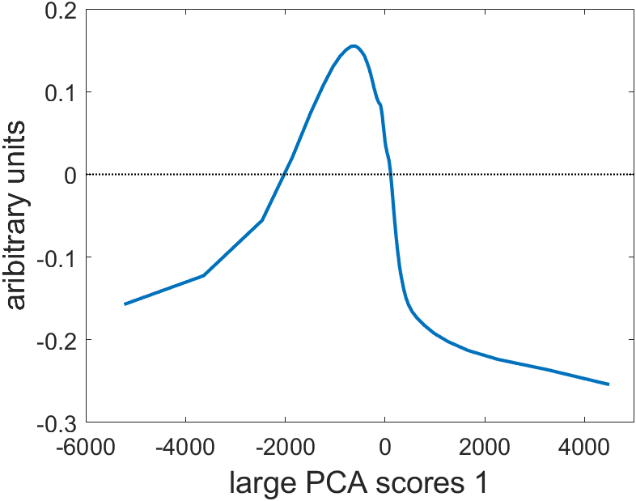

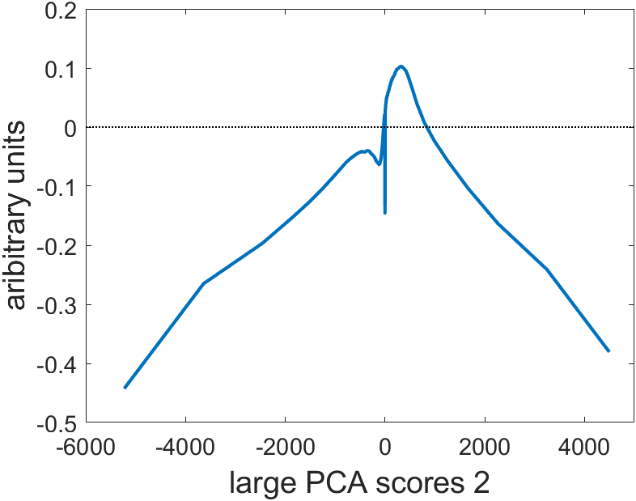


Pixel score distributions of large PCA component 3. Principal Component Analysis. Top: Scatter plots of components 1-2 accounting for 54.3, 25.8, % of the total variance Bottom: loadings of components 1 and 2.


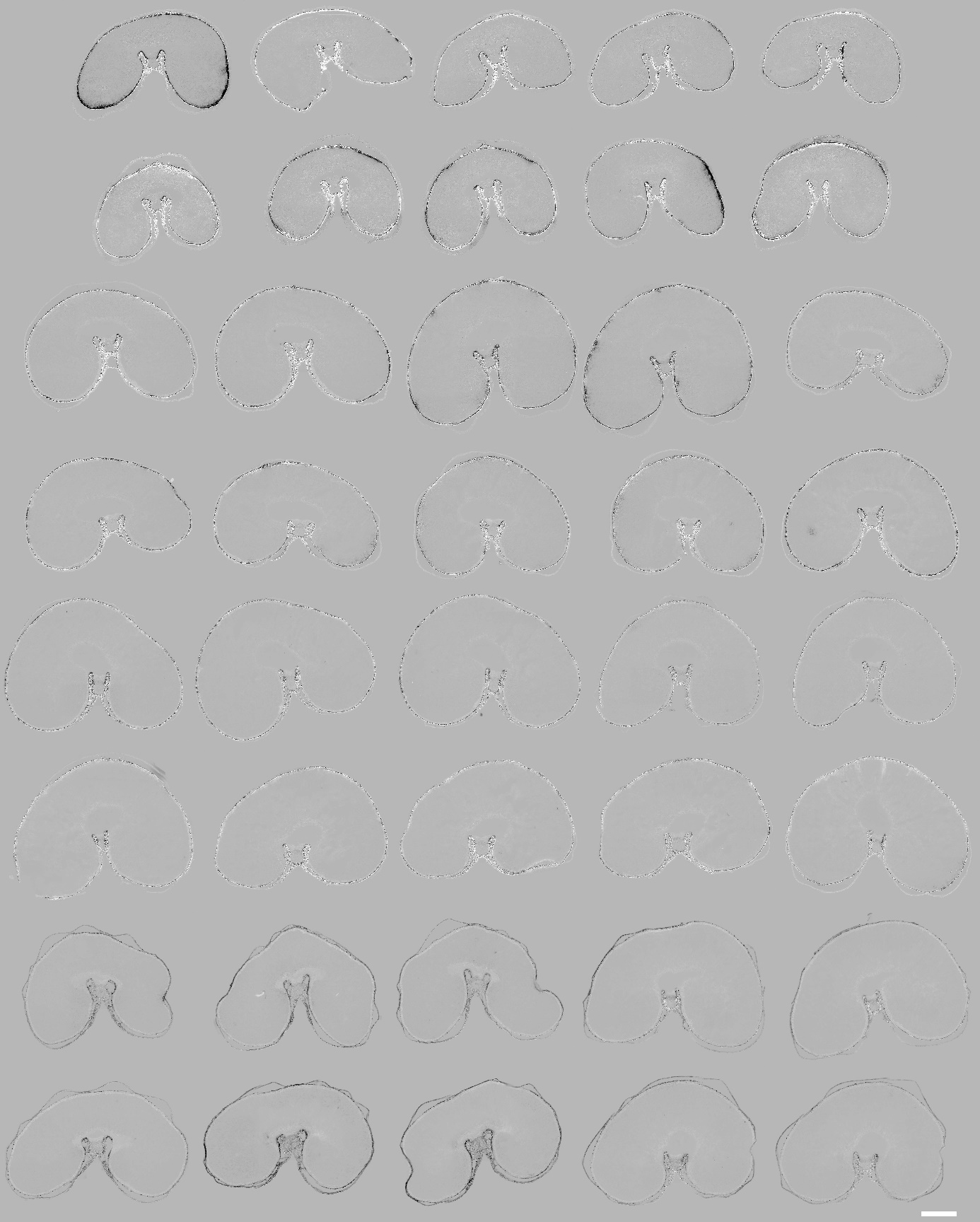


Large PCA. Principal Component 3. Montages of the 40 score images of the series. Sections for stages 250°DAF (lines 1 and 2), 450°DAF (lines 3 and 4), 650°DAF (lines 5 and 6) and 850°DAF (lines 7 and 8), respectively. intensities can be compared. Scale bar represents 1 mm.


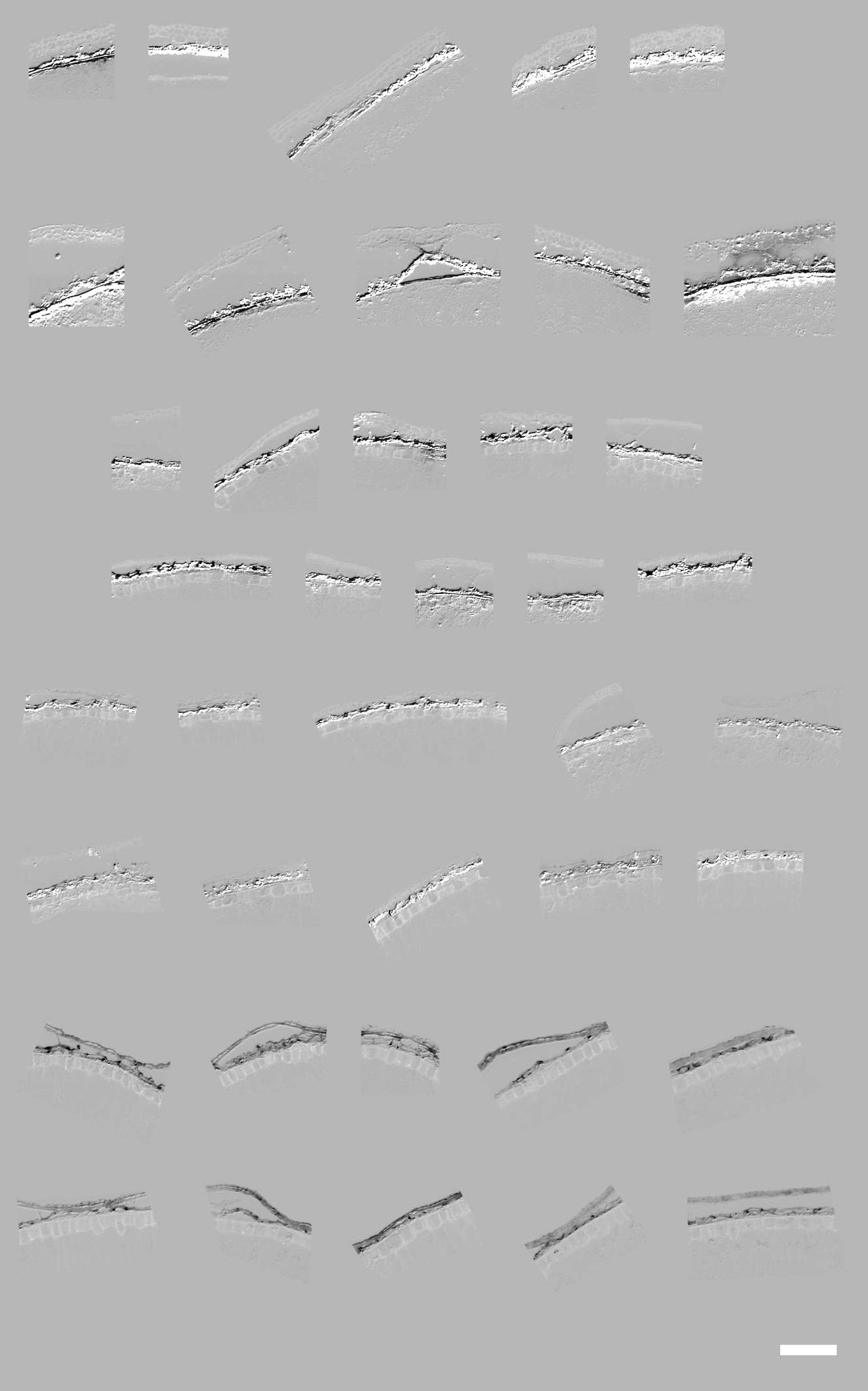


Large PCA. Principal Component 3. Zoom of the dorsal region. Montages of the 40 score images of the series. Sections for stages 250°DAF (lines 1 and 2), 450°DAF (lines 3 and 4), 650°DAF (lines 5 and 6) and 850°DAF (lines 7 and 8), respectively. intensities can be compared. Scale bar represents 250 µm.


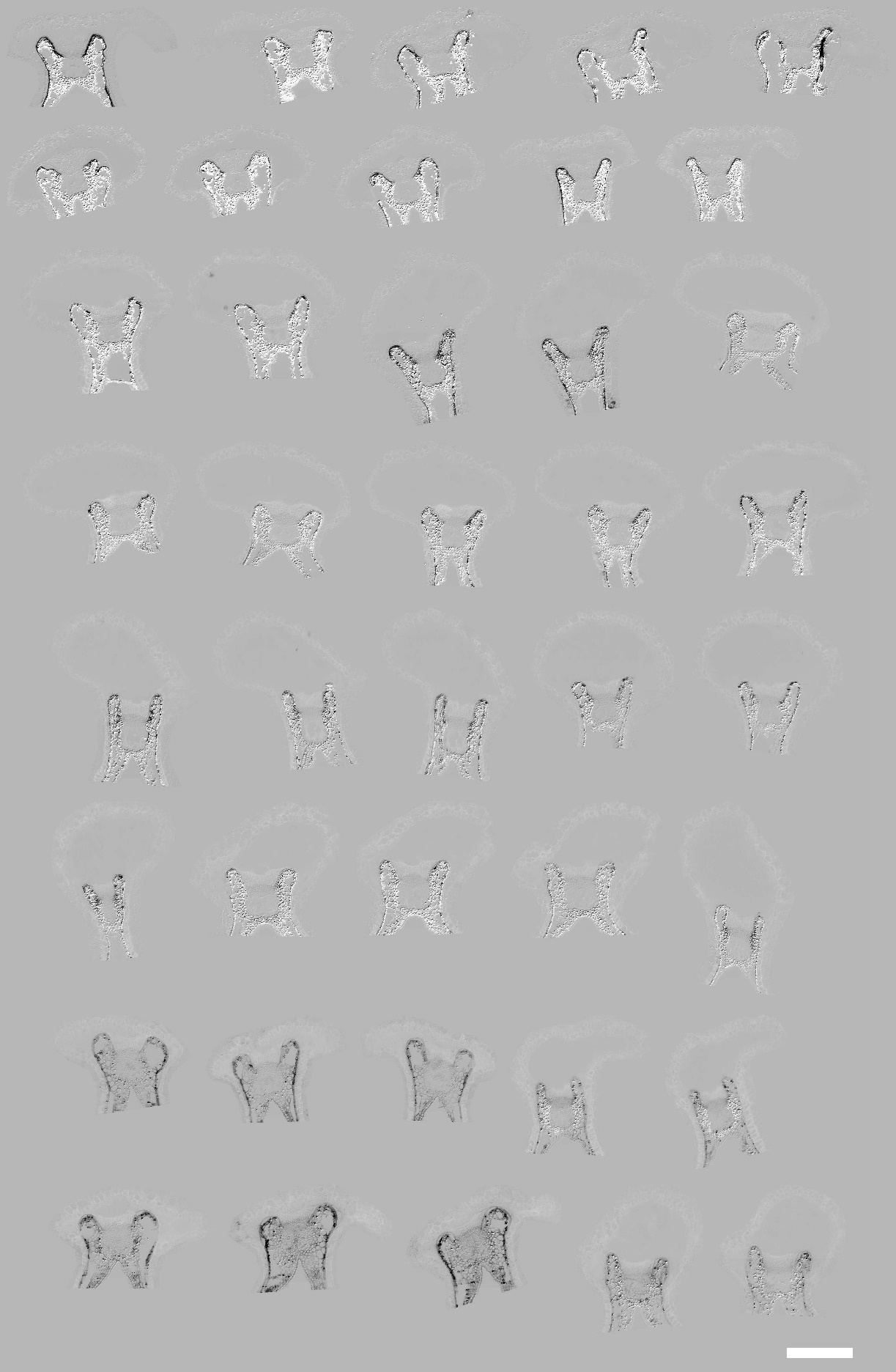


Large PCA. Principal Component 3. Zoom of the crease region. Montages of the 40 score images of the series. Sections for stages 250°DAF (lines 1 and 2), 450°DAF (lines 3 and 4), 650°DAF (lines 5 and 6) and 850°DAF (lines 7 and 8), respectively. intensities can be compared. Scale bar represents 750 µm.
