# Supplementary material for: Exploring the structural variability in developing wheat grains using autofluorescence multispectral imaging at the macroscopic scale
Source: Front Plant Sci. 2025 Jun 19;16:1580426. doi: 10.3389/fpls.2025.1580426 (PMC12222247; doi:10.3389/fpls.2025.1580426)
Supplement: Supplementary file 1 [file DataSheet1.zip › SupplementaryData/SupplementaryTable2.docx]

**Supplementary Table 2** Summary of characteristics, tissue distribution and tentative attribution of large PCA components

|  | **stage of development** | | **250°DAF** | **450°DAF** | **650°DAF** | **850°DAF** |  |
| --- | --- | --- | --- | --- | --- | --- | --- |
|  | component | % var |  |  |  |  | tentative attribution |
| General | **CP1** Red emission after excitation Blue (BL) and Green (GR) | 82.26% | + + + + fluorescence intensity | + + | + + /less homogeneous | + /dots | **chlorophyll** |
| Tissues |  |  | mainly in endocarp and crease mesocarp | mainly in endocarp and crease mesocarp | mainly in endocarp and crease mesocarp | mainly in endocarp and crease mesocarp |  |
| General | **CP2** mainly black pixels with negative scores Blue emission after excitation in UV1 (U1) and UV2 (U2), white pixels with positive scores Red emission after excitation in BL | 13.78% | + mainly UV fluorescence (and a little red fluorescence ) | + + | + + | + | (red emission : chlorophyll), **UV : phenolics** (hydroxycinnamic acids and lignin) |
| Tissues |  |  | red fluorescence in endocarp and crease mesocarp, UV fluorescence in cell walls of outer pericarp, nucellus epidermis, nucellar projection, vascular tissues, aleurone | UV fluorescence in cell walls of outer pericarp, nucellus epidermis, nucellar projection, vascular tissues, aleurone | UV fluorescence in cell walls of outer pericarp, nucellus epidermis, nucellar projection, vascular tissues, aleurone | UV fluorescence in cell walls of outer pericarp (but decreased), nucellus epidermis, in the gel of the apoplastic cavity, and nucellar projection, and aleurone most fluorescent tissues |  |
| General | **CP3** Red emission after GR excitation (black negative scores) and BL excitation (white positive scores) | 2.02% | positive (BLr) + + dominant in the crease, negative (GRr) heterogeneous | positive + | positive +/-, negative pixels as dots | less positive pixels, negative signal more homogeneous + | **flavonoids?** |
| Tissues |  |  | positive in endocarp and mesocarp, negative in the testa | endocarp black dots, negative in the testa, while most of other tissues are white (positive signal) | mainly positive signal in mesocarp, and endocarp, negative signal as dots | outer pericarp negative signal, dots in endocarp and crease mesocarp |  |
| General | **CP4** Blue emission after U1 excitation (white positive scores), Green emission after BL excitation (black negative scores) | 1.3% | mainly white pixels positive scores | mainly white pixels positive scores | heterogeneous | mainly black pixels negative scores | negative signal **lignin** |
| Tissues |  |  | all outer tissues | positive signal for outer tissues, negative signal in the pigment strand and,as dots,in endocarp cells | positive signal for aleurone, nucellar projection, negative dots for endocarp and mesocarp, negative signal in pigment strand | negative signal in cell walls of pericarp, pigment strand, and vascular tissues, positive signal in cell walls of the aleurone and modified aleurone, in nucellar projection and in the cavity gel |  |
| General | **CP5** Blue emission after U1 excitation (positive), or U2 excitation (negative) | 0.51% | mainly white pixels with positive scores UV1, a little black pixels with negative scores | a little more black pixels | a little more black pixels, aleurone cell walls mainly white pixels | mainly white pixels with positive scores with some black pixels | **phenolics** |
| Tissues |  |  | positive pixels in outer layers, negative signal in the outer pericarp | a little more negative signal in the pericarp, aleurone cell walls mainly positive signal but in lobe region negative signal in anticlinal cell walls, epicarp cell walls in the crease region negative signal | negative signal in the outer pericarp and in anticlinal cell wall of the aleurone while positive signal in synclinal cell walls. In the crease region positive pixel in the modified aleurone cell walls, in nucellar projection and nucellar epidermis. White (positive) dots and black (negative) dots in the crease mesocarp, and negative pixels in the cell walls of the epicarp | Heterogeneous, more positive pixels, aleurone anticlinal cell wall negative signal, in the crease positive pixels in the pericarp |  |
