# Supplementary figures and images for: Exploring the structural variability in developing wheat grains using autofluorescence multispectral imaging at the macroscopic scale
Source: Front Plant Sci. 2025 Jun 19;16:1580426. doi: 10.3389/fpls.2025.1580426 (PMC12222247; doi:10.3389/fpls.2025.1580426)

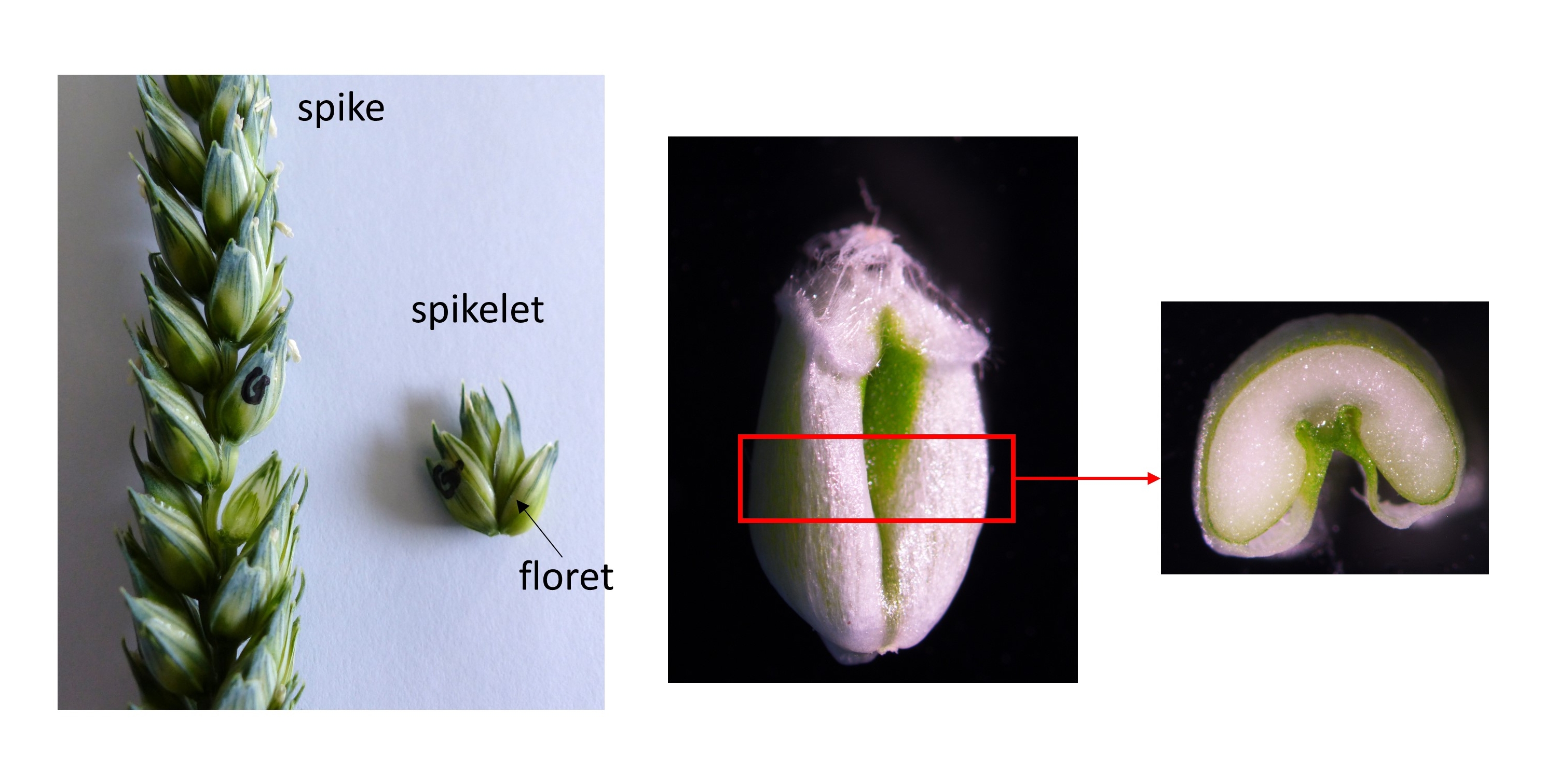

Supplement: Supplementary file 1 [file DataSheet1.zip › SupplementaryData/Supplementary Figure01.jpg]

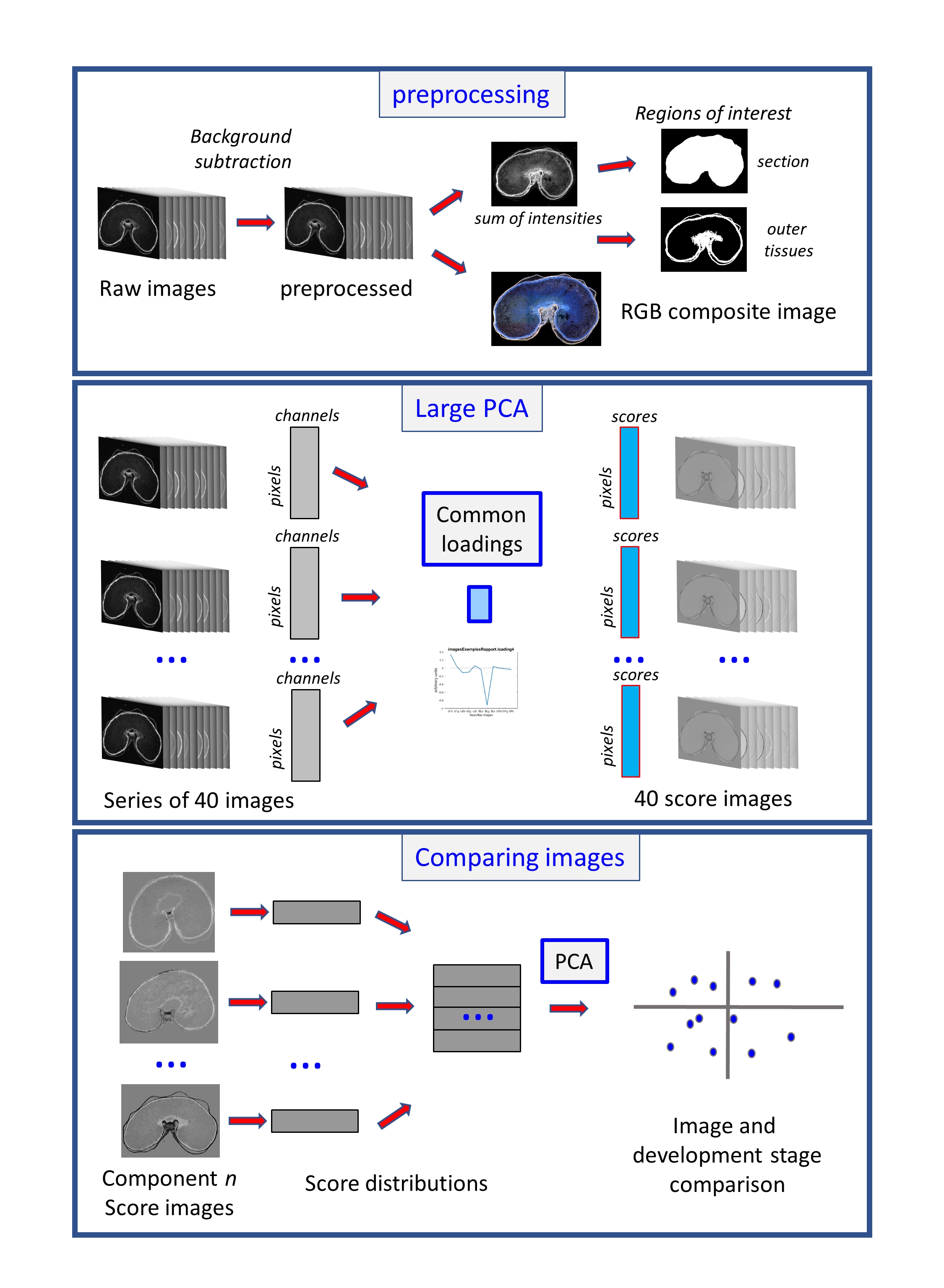

Supplement: Supplementary file 1 [file DataSheet1.zip › SupplementaryData/SupplementaryFigure02-Workflow.jpg]

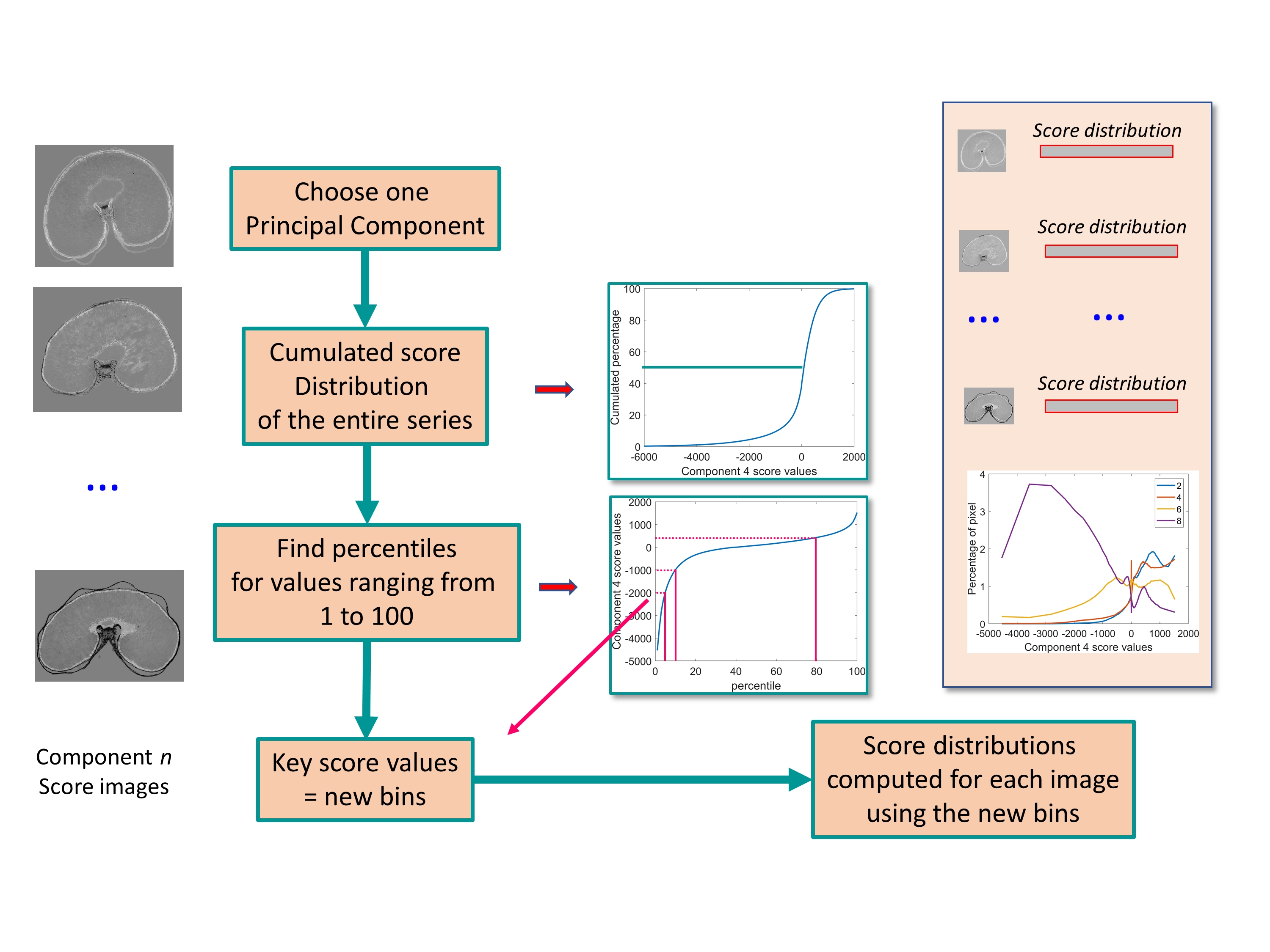

Supplement: Supplementary file 1 [file DataSheet1.zip › SupplementaryData/SupplementaryFigure03-ScoreDisribution.jpg]

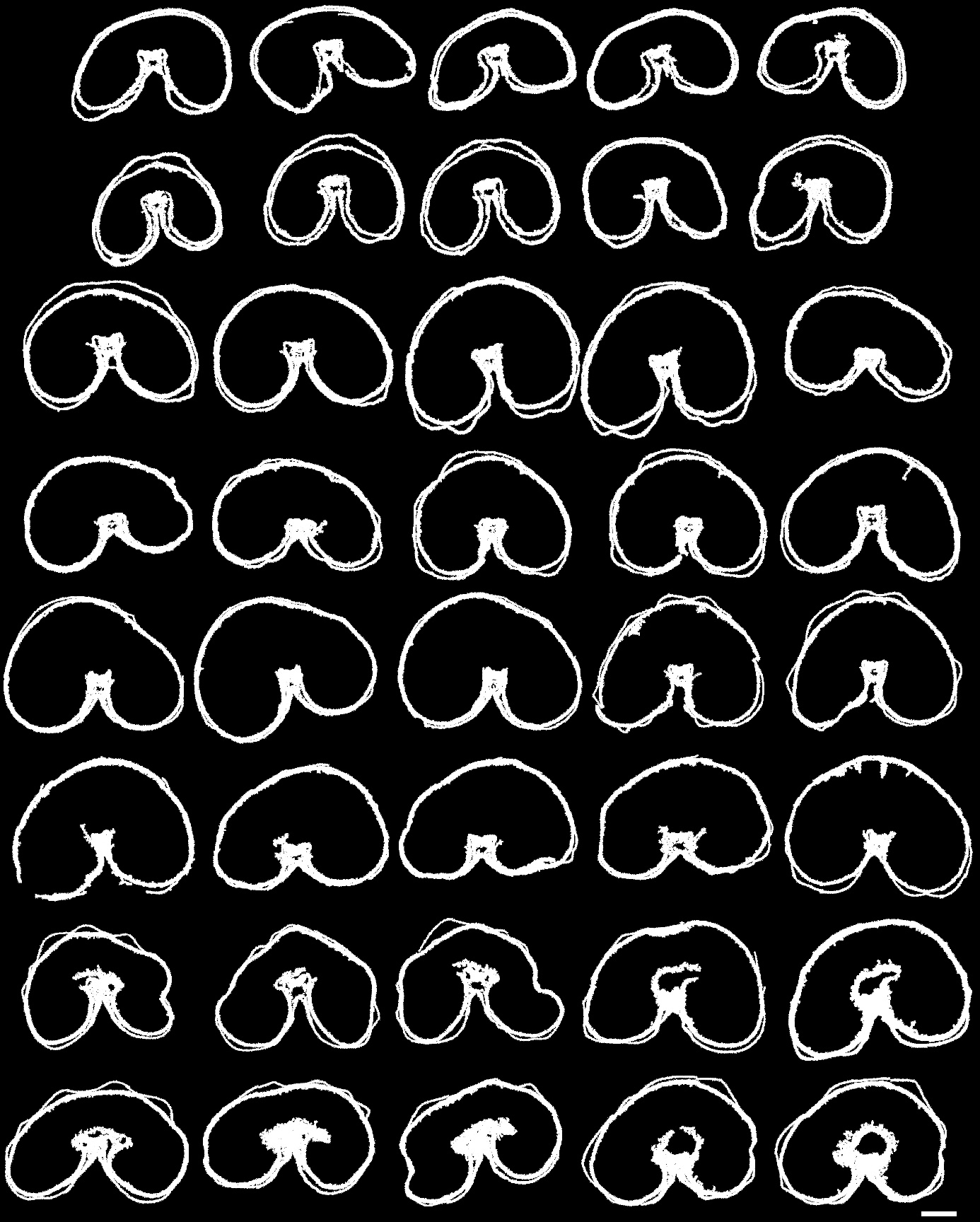


Region of interest of outer tissues. Scale bar represents 1 mm.

Supplement: Supplementary file 1 [file DataSheet1.zip › SupplementaryData/SupplementaryRoiOuterTissues.docx]
